# Tropical origins of the recent trends in Northern Hemisphere wintertime jet streams

**DOI:** 10.1038/s41467-026-74980-3

**Published:** 2026-06-29

**Authors:** Gwendal Rivière, Sébastien Fromang, Sebastian Schemm

**Affiliations:** 1 LMD/IPSL, CNRS, Ecole Normale Supérieure-PSL Université, Sorbonne Université, Ecole Polytechnique-Institut Polytechnique de Paris, Paris, France; 2 LSCE/IPSL, CEA, CNRS, UVSQ, Université Paris-Saclay, Gif-sur-Yvette, France; 3 Department of Applied Mathematics and Theoretical Physics, University of Cambridge, Cambridge, UK

**Keywords:** Climate change, Atmospheric dynamics

## Abstract

Mid-latitude jet streams are fundamental to global weather and influence high-impact meteorological events. Climate models consistently project a poleward shift of the jets under anthropogenic warming and evidence for such a shift recently emerged in reanalysis data. Here we show that trends in Northern Hemisphere winter jet streams over the past 45 years are closely linked to the increased number of intense mid-latitude precipitation events over a large area in the western North Pacific, themselves favored by tropical precipitation anomalies associated with the more recurrent La-Niña-like conditions but also during recent El-Nino seasons when the Madden Julian Oscillation is under phases 4 to 6. During these events, the North Pacific jet is accelerated on its poleward flank followed by an acceleration of the North Atlantic jet. The findings reveal that tropical Pacific precipitation trends, through their modulation of quasi-stationary and synoptic eddy activity, play a pivotal role in shaping recent mid-latitude jet stream trends in the Northern Hemisphere. This provides an avenue of research to evaluate climate models and their ability to reproduce historical and future trends.

## Abstract (web summary)

This study shows that the recent trends in the North Pacific and the North Atlantic jet streams in winter are linked to an increased number of intense North Pacific precipitation events, which are in turn favored by tropical Pacific precipitation trends.

## Introduction

The last four decades of reanalysis covering the satellite area period (e.g., since 1979) exhibit a significant trend toward a poleward shift and an upward lift of mid-latitude zonal-mean jets and storm tracks^1,2^, which was not as clear in previous studies covering shorter periods^3,4^. During December-February (DJF) the poleward shift is visible in both hemispheres on the zonal average but is more pronounced in the Southern Hemisphere (SH). Such a general trend is absent in the summer Northern Hemisphere (NH). These observed trends in the jet streams positions may originate from internal variability or various anthropogenic forcings (changes in ozone, aerosols, CO_2_). However, due to the growing forcing signals and the lengthening of the observations period, it is now easier to attribute observed trends to various forcing signals by running climate models with historical forcings^5^. As a result, it is now well established that the poleward shift of the SH jet streams until the early 2000s was due to both an increase in CO_2_ concentration and to ozone depletion and that the shift was recently attenuated due to ozone recovery^6,7^.

The poleward shift of the jets is a robust response to increased CO_2_ in future climate simulations^8,9^. It is more pronounced in the SH than in the NH where it depends on the season^10^. It has been confirmed by multiple sensitivity numerical experiments since the pioneering work of ref. ^11^ until the most recent Coupled Model Intercomparison Projects^12–17^.

Many mechanisms have been proposed to explain the poleward-shifted jets in response to increased CO_2_, but there is currently no consensus^18^. Several mechanisms rely on the increased upper-tropospheric temperature gradient in presence of amplified tropical warming^19,20^, which induces a poleward shift of the jet. In that scenario, the weaker poleward-shift response observed in the NH compared to the SH is accounted for by Arctic warming, which decreases the low-level temperature gradient in the mid-latitudes, partly compensating the aformentioned poleward shift^19,20^.

Despite these successes in attributing and explaining the mid-latitude poleward-shifted jets associated with anthropogenic forcings, uncertainties and discrepancies remain when comparing observations and reanalysis with climate models^5^. In particular, coupled and atmospheric-only climate models struggle to reproduce as intense historical trends as in the reanalyzes like the increased storminess in the SH^21^ and the strength of the poleward-shifted jets in the NH during winter^1,22,23^. The latter is quite surprising because coupled models display a stronger tropical warming than in observations and reanalyzes^24,25^. It questions about the sensitivity of the jet poleward shift amplitude to the upper-tropospheric temperature gradient described above^19,20^. Therefore although the models are able to qualitatively reproduce the zonal-mean observed jet streams trends, there is large intermodel spread^26^ and the models do not necessarily reproduce the poleward shifts with the same intensity and the same mechanistic reasons as in the observations^1,5^. Coupled models are even less accurate than atmospheric-only models in reproducing observed trends jet streams trends. Part of the reason is their inability to simulate the La-Niña like sea surface temperature (SST) trends^21,27–29^ and consequently the associated tropics-extratropics teleconnections^30^. This motivates the present study which aims at investigating the jet streams trends in the NH during winter (DJF) using reanalysis data only, without relying on model results and focusing on tropical origins.

## Results

### Trends in jets, storm-tracks and precipitation

**Fig. 1:**
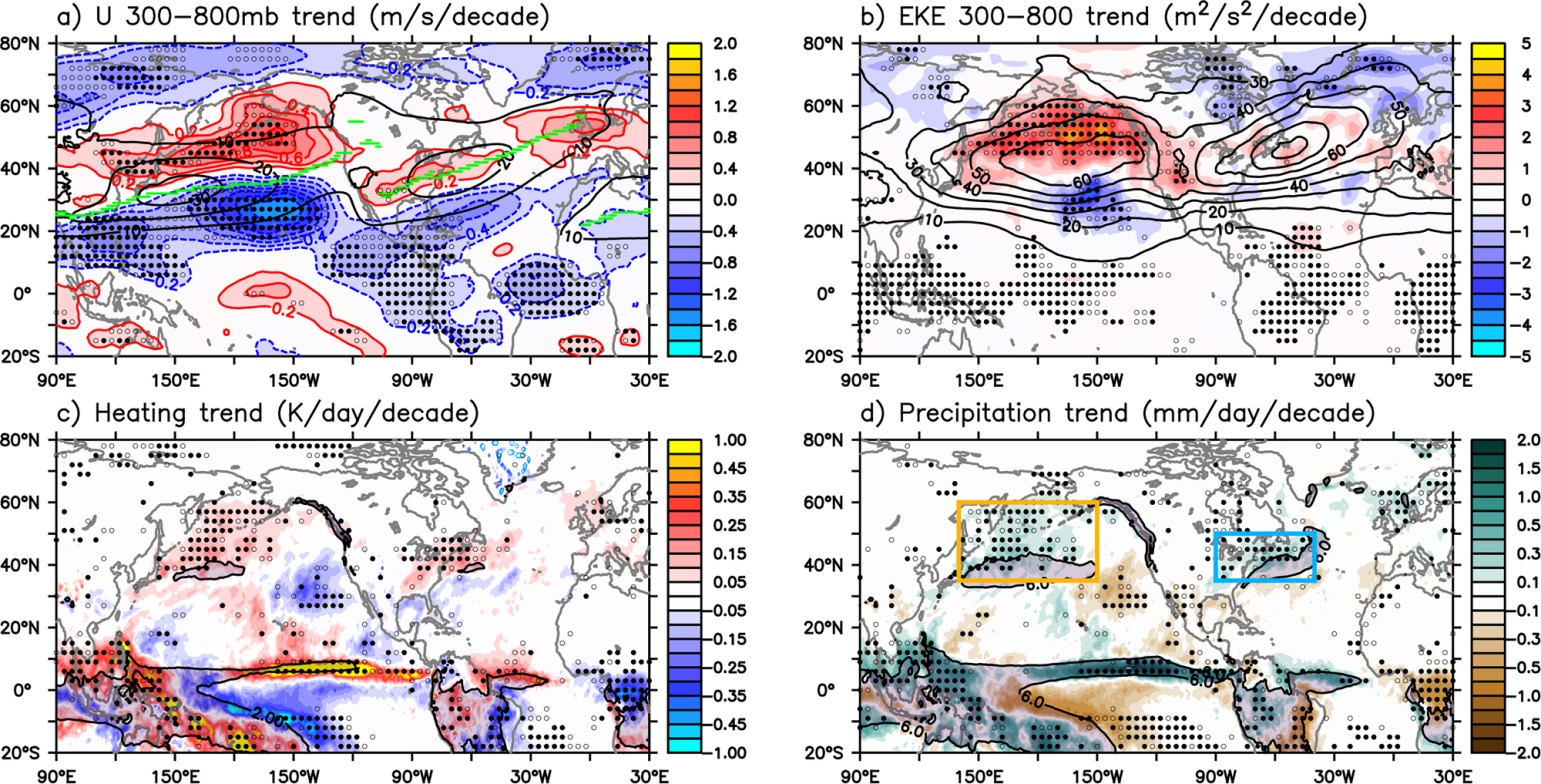
Least squares linear regression trends during December-January-February (DJF) from 1979 to 2024 using ERA5 reanalysis. **a** 300–800 hPa zonal wind (int: 0.2 m s^−1^ decade^−1^; red shadings for positive values and blue shadings for negative values) and climatology (contours). The green line corresponds to the climatological jet axis. **b** 300–800 Eddy Kinetic Energy (EKE) (shadings; int: 0.5 m^2^ s^−2^ decade^−1^) and climatology (contours; int: 10 m^2^ s^−2^). **c** 300–800 hPa heating rate (shadings) and climatology (contours values greater than 2 K day^−1^). **d** total precipitation (shadings; units mm day^−1^ decade^−1^) and climatology (black contour and shaded for values greater than 6 mm day^−1^). Black dots and open circles indicate significance at the 5% and 10% levels respectively using a two-sided t-test.

Figure 1 shows DJF linear trends in different variables for 1979–2024 and ERA5 reanalysis. Looking at the vertically-averaged zonal wind trends (Fig. 1a), we observe a strong significant poleward shift of the North Pacific (NPac) jet, consistent with previous studies^23,31^. The North Atlantic (NAtl) jet is accelerated along its axis extending from the US east coast to northwestern Europe, but decelerates on its flanks. Although the positive trend of the jet speed is not significant its reduction on the flanks is significant over large areas. Such an acceleration has been already detected over the same period^32,33^ and over a longer period (1951–2020)^22^. It has been shown not to result in the reanalysis from assimilation increment issues^32^.

In the NPac, the vertically-averaged eddy kinetic energy (EKE) trend shows both a poleward shift and an increase with significant values at 95% (Fig. 1b). In the NAtl, there is a slight strengthening in the peak of the climatological areas and a decrease on their flanks. The strengthening of the Atlantic storm track is not much significant when looking at vertical averages (Fig. 1b) but is significant at lower levels like 800 hPa (Fig. S1).

Global warming is expected to result in increase in latent heating and precipitation within storm-tracks^34,35^. In mid-latitudes, diabatic heating and precipitation increase significantly north of their climatological means and at the entrance of the storm-track regions in both ocean basins (see orange and blue boxes in Fig. 1c, d). In the eastern NPac, there is also a significant decrease between 25°N and 40°N and between 150°W and 120°W. In the tropics, there is a significant increase in heating and precipitation over the maritime continent and a westward shift of the patterns over the South Pacific Convergence Zone (SPCZ), consistent with the La-Niña-like trend and the strengthening of west-to-east SST gradient^29,36^. We also observe significant positive trends over the Inter-Tropical Convergence Zone (ITCZ) in the Central Pacific and Central Atlantic.

Precipitation is a small-scale, intermittent variable that is a key component of the water cycle. However, it is difficult to observe, particularly over oceans. It is thus common practice to conduct intercomparisons between different precipitation datasets to assess the reliability of the results^37,38^. The last IPCC report mentioned an increase in mid-latitude precipitation since the 1980s over land with medium confidence, while estimates of the trends over ocean are uncertain. To assess the uncertainties of ERA5 precipitation trends, Figs. S2 to S4 compare seven distinct precipitation products, three reanalysis, three observations-derived (satellite and/or gauge) datasets and a multi-source dataset (see methods and supplement information). Our conclusion is that ERA5 precipitation trends resemble those of other reanalysis and are included within the observational precipitation uncertainty range.

### Contribution of North Pacific and North Atlantic precipitation to jet stream trends

To investigate the link between precipitation and jet trends, two areas of significant positive trends in ERA5 precipitation are defined in the NPac and the NAtl, and are hereafter denoted as the PAC and ATL regions (Fig. 1d). The seasonal-mean precipitation is averaged over each of these regions to get two normalized precipitation indexes denoted as *I*_PAC_ and *I*_ATL_ respectively. By regressing the seasonal-mean zonal wind on *I*_PAC_ and *I*_ATL_ and then multiplying the obtained regression coefficients by the trends in *I*_PAC_ and *I*_ATL_, we deduce the zonal wind trends congruent with *I*_PAC_ and *I*_ATL_ trends respectively. This is similar to what was done to determine the contribution of Southern Annular Mode trends to the SH jet stream trends^39^. For the zonal wind, this can be formally written as

1 $${{\mathcal{T}}}({\hat{u}}_{PAC})={\beta }_{1PAC}^{u}{{\mathcal{T}}}({I}_{PAC})\\ {{\mathcal{T}}}({\hat{u}}_{{{\rm{ATL}}}})={\beta }_{1\,{\mbox{ATL}}\,}^{u}{{\mathcal{T}}}({I}_{{{\rm{ATL}}}}),$$

where ${{\mathcal{T}}}$ denotes the trend, ${\hat{u}}_{PAC}$ and ${\hat{u}}_{{{\rm{ATL}}}}$ are the zonal wind estimates deduced from the regressions of the zonal wind *u* on *I*_PAC_ and *I*_ATL_, respectively, while ${\beta }_{1PAC}^{u}$ and ${\beta }_{1\,{\mbox{ATL}}\,}^{u}$ represent the slopes of the regressions.

**Fig. 2:**
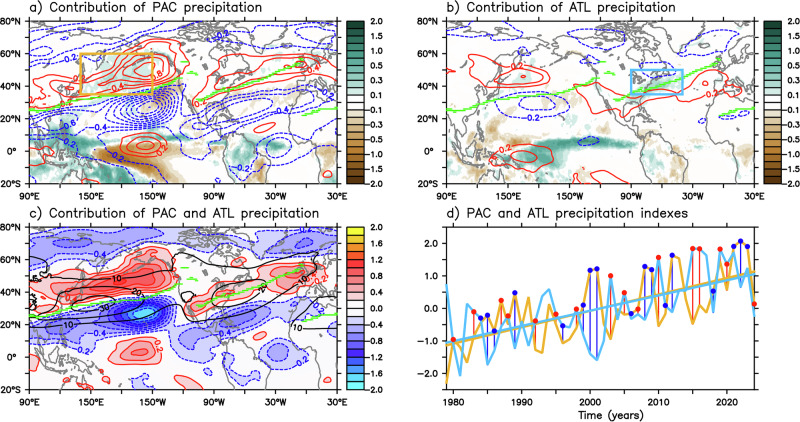
Zonal wind and precipitation linear trends congruent with mid-latitude precipitation trends over two specific regions in the North Pacific and North Atlantic and relationship with El-Niño Southern Oscillation (ENSO). **a** Contribution of precipitation in PAC (Pacific region delimited by the orange box) to u (contours) and precipitation (shading) trends. **b** Same as **a** but for precipitation in ATL (Atlantic region delimited by the blue box). **c** same as **a** for u but using the PAC and ATL multilinear regression (red shadings for positive values and blue shadings for negative values) and climatology (contours). **d** PAC and ATL indexes as function of time. Blue and red dots correspond to Nino34 index greater than 1 and less than −1, respectively. The black line corresponds to the climatological jet axis.

The zonal wind trend estimated from the regression on *I*_PAC_, that is ${{\mathcal{T}}}({\hat{u}}_{{{\rm{PAC}}}})$, closely resembles the observed trend in zonal wind (colored contours in Figs. 1a and 2a) with a spatial correlation of 0.91. The signs and amplitudes agree very well, and both the poleward-shifted NPac jet and the accelerated NAtl jet are recovered in the trend deduced from the regression on *I*_PAC_. In contrast, the estimated trend from the regression on *I*_ATL_ does not resemble much the zonal wind trend (Figs. 1a, 2b), the spatial correlation being 0.48. ${{\mathcal{T}}}({\hat{u}}_{{{\rm{ATL}}}})$ exhibits a slight poleward-shifted pattern over the western NPac and an equatorward-shifted pattern in the whole NAtl, which contrasts with the observed wind trends. To conclude, the NPac jet trend is directly linked to NPac precipitation trend, while local precipitation events in NAtl seem to contribute much less to the NAtl jet trend. Of course, such a regression analysis does not provide causal relationship, it is just there to show how much of the zonal wind trends are accompanied by precipitation trends in various regions. Mechanistic arguments will be provided later in section 5.

Following the same strategy, we can estimate the combined contribution of the PAC and ATL precipitation to the zonal wind trends by computing the multiple linear regression coefficients based on the two indicators *I*_PAC_ and *I*_ATL_:

2 $${{\mathcal{T}}}({\hat{u}}_{{{\rm{PAC}}}{\&}{{\rm{ATL}}}})={\beta }_{1{\mbox{PAC}}/{\mbox{ATL}}\,}^{u}{{\mathcal{T}}}({I}_{{{\rm{PAC}}}})+{\beta }_{1{\mbox{ATL}}/{\mbox{PAC}}\,}^{u}{{\mathcal{T}}}({I}_{{{\rm{ATL}}}}),$$

where ${\beta }_{1\,{\mbox{PAC}}/{\mbox{ATL}}\,}^{u}$ and ${\beta }_{1\,{\mbox{ATL}}/{\mbox{PAC}}\,}^{u}$ are the partial regression coefficients with the two indicators *I*_PAC_ and *I*_ATL_ respectively. The resulting trend ${{\mathcal{T}}}({\hat{u}}_{{{\rm{PAC}}}{\&}{{\rm{ATL}}}})$ (Fig. 2c) is similar to the trend derived from the regression on *I*_PAC_, that is ${{\mathcal{T}}}({\hat{u}}_{{{\rm{PAC}}}})$, and resembles the observed zonal wind trend very well (spatial correlation of 0.97). Interestingly, ${{\mathcal{T}}}({\hat{u}}_{{{\rm{PAC}}}{\&}{{\rm{ATL}}}})$ is very close to the sum of ${{\mathcal{T}}}({\hat{u}}_{{{\rm{PAC}}}})$ and ${{\mathcal{T}}}({\hat{u}}_{{{\rm{ATL}}}})$, which is expected because the two indexes *I*_PAC_ and *I*_ATL_ have a weak and non-significant correlation coefficient (0.19). Despite the large dominance of the Pacific contribution, there are regions where the ATL contribution matters. For instance, over North America and the western NAtl, the contribution of PAC precipitation (Fig. 2a) has positive (resp. negative) tendencies north (resp. south) of the jet axis, but the contribution of ATL precipitation (Fig. 2b) exhibits the opposite pattern. When using the multiple linear regression with two indicators *I*_PAC_ and *I*_ATL_, the zonal wind trend pattern better resembles the observed trend of an accelerated jet in the NAtl (compare Fig. 2c with Fig. 1a).

The same analysis has been made by regressing the precipitation seasonal means onto *I*_PAC_ and *I*_ATL_ (shadings in Fig. 2a, b). As for the zonal wind, a large part of the precipitation trend can be reconstructed using the regression onto *I*_PAC_: the westward shift of the precipitation in the tropical Pacific and the increased precipitation over the whole maritime continent typical of La Niña events, together with increased precipitation in Central America and tropical Atlantic. Only two precipitation trend patterns are better reproduced by the regression onto *I*_ATL_: the increased precipitation in the Central Pacific near 5°N, which resembles more the El-Niño events and, by construction, the increased precipitation in the western NAtl near 40°N.

### Links with ENSO and the MJO

The two regression coefficients of precipitation onto *I*_PAC_ and *I*_ATL_ have opposite signs in the tropical Pacific and their patterns seem to project onto La-Niña and El-Niño events respectively (shadings in Fig. 2a, b). This is confirmed in Fig. 2d. Although *I*_PAC_ and *I*_ATL_ have both positive trends, they are anticorrelated on interannual time scales. When the data is detrended, the correlation is −0.39 which is significant at 99% level. High values of *I*_PAC_ are reached during La-Niña events like 1988–1989, 1999–2000, 2007–2008 or more recently 2021–2022. In contrast, high values of *I*_ATL_ are reached during El-Nino events like 2002–2003, 2009–2010, 2015–2016.

To further investigate the links between mid-latitude precipitation trends and tropical modes of variability like ENSO and MJO, sustained intense precipitation events in the mid-latitude PAC and ATL regions are selected (see methods). In what follows, only the PAC events are discussed. Equivalent figures for the ATL events are presented in the supplement (Figs. S6, S7, S8). As expected from the trends in Fig. 1, the probability density function of the low-frequency (periods greater than 10 days) precipitation in the PAC region is shifted toward higher values in the recent period (2002–2024 in red) compared to the older period (1979–2001 in blue). We selected all the events beyond 1.5 standard deviation of the low-frequency precipitation; there are 2.5 times more events in the recent period than in the old period (54 events during 2002–2024 versus 20 during 1979–2001). This ratio decreases to a factor of 1.6 when the low-pass filter is not applied. This decrease indicates that it is the number of sustained and intense precipitation events that differs most between the two periods and not so much the number of short and intense precipitation events.

**Fig. 3:**
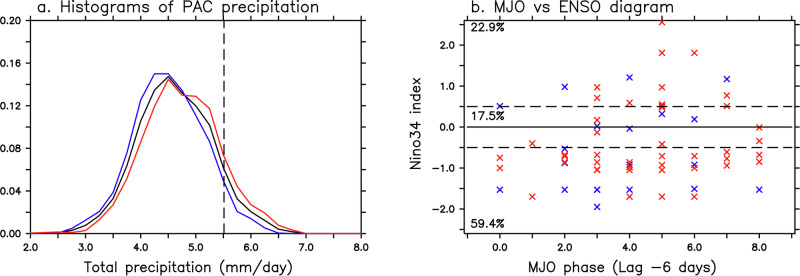
Selection of sustained heavy precipitation events in the North Pacific and links with Madden Julian Oscillation (MJO) and El-Niño Southern Oscillation (ENSO). **a** Histograms of the low-frequency total precipitation in PAC (Pacific region delimited by the orange box in Fig. 2a) for the 1979–2001 (blue) and 2002–2024 (red) periods. **b** Nino34 index versus MJO phase at lag −6 days for the 74 sustained heavy precipitation events in the PAC region (orange box in Fig. 2a), 20 of them belong to the 1979–2001 period (blue) and 54 events to the 2002–2024 period (red). The vertical dashed line in **a** corresponds to the 1.5 standard deviation value.

Figure 3b shows the distribution of these 74 intense and sustained precipitation events in the PAC region as a function of ENSO and MJO phases. Sustained precipitation events in the PAC region tend to occur during La Niña-affected months (58.5%) independent of the MJO phase and during neutral or El Niño-affected months (41.5%) when MJO varies between phase 4 to 6. Both situations (La Niña or MJO around phases 4 to 6) are characterized by more precipitation over the maritime continent, consistent with Fig. 2a.

### Mechanism

**Fig. 4:**
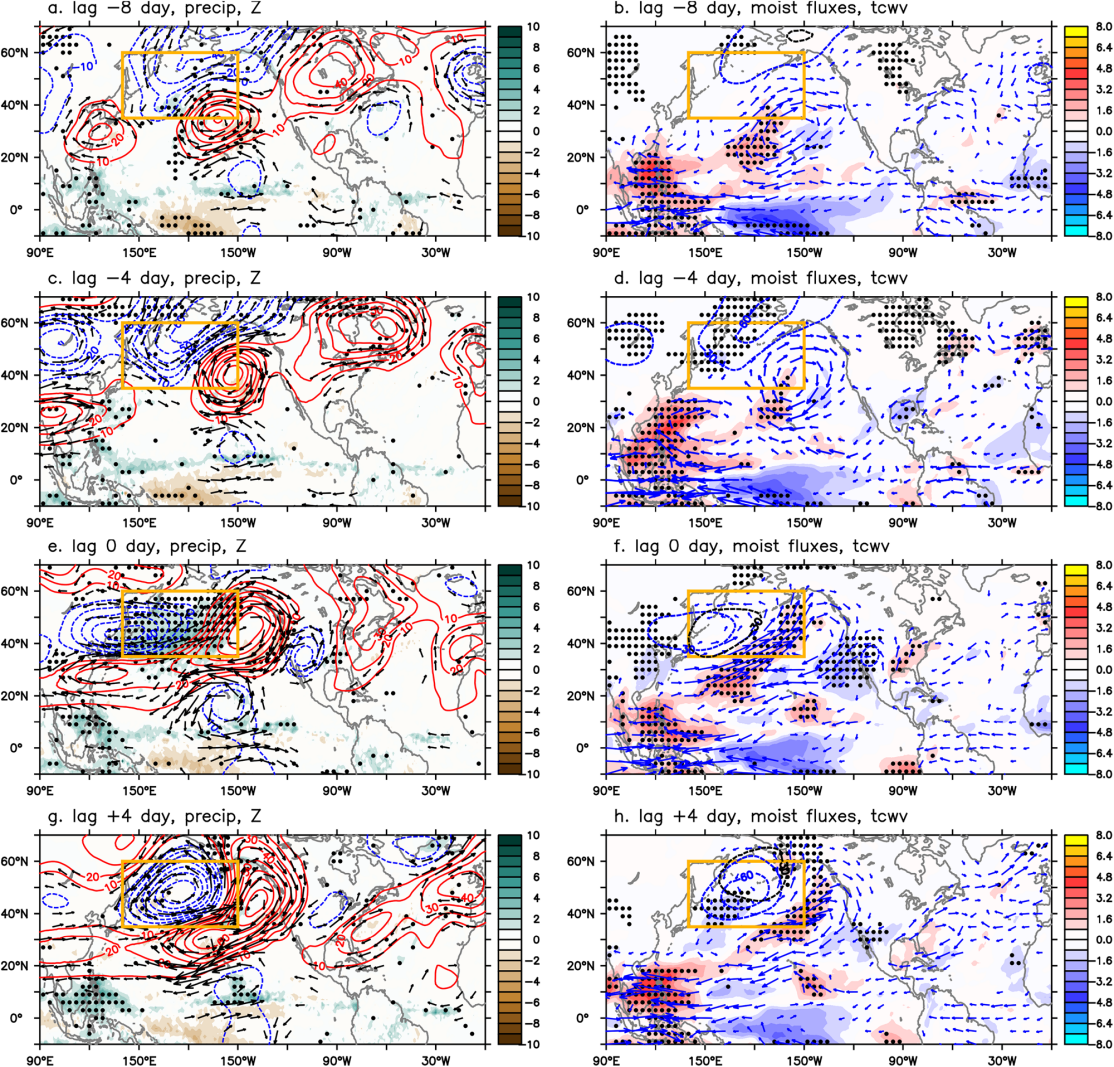
Time-lag anomalous composites of geopotential, precipitation and moisture for intense precipitation events in PAC (Pacific region delimited by the orange box). (left column) total precipitation (shadings; units: mm/day) and geopotential height (Z) at 300 hPa (int: 10 m; red and solid contours for positive values and blue and dashed for negative values) and (right column) total column water vapor (tcwv; shadings; units: kg m^−2^), moisture fluxes at 800 hPa (blue vectors for values of the magnitude larger than 6 × 10^−3^ kg/kg m/s) and the geopotential height at 300 hPa (values less than −30 m; int: 30 m; blue and dashed contours) and 800 hPa (values less than −30 m; int: 30 m; black and dashed contours). Each quantity is averaged over 3 days and centered at the indicated lag. Black dots indicate significance at the 5% level using a t-test for the precipitation on the left column and the total column water vapor on the right column. Time lags are **a**, **b** −8 days, **c**, **d** −4 days, **e**, **f** 0 day, **g**, **h** +4 days.

This section addresses the question of how the tropical precipitation anomalies of La-Niña type or MJO-phases 4 to 6 impact precipitation events in the storm-track entrance region (PAC) and the mid-latitude jets. This is done using lagged composites of the 74 events. Eight days prior to the PAC events (Fig. 4a), two trough anomalies are visible on both sides of the Equator near 150°W. In addition, precipitation is reduced at the equator near 180°W. Such anomalies are reminiscent of La-Niña and MJO phases 4-to-6 situations^40,41^. Further north, a ridge anomaly is present on the southeastern side of the PAC region and a trough anomaly on the northern side. This succession indicates the presence of a quasi-stationary Rossby wave emitted from tropical convection anomalies in the central Pacific^42^. The Rossby wave train leads to an intensification of the upper-tropospheric westerlies in the PAC region. Since the latter region is located north of the jet axis (Fig. 2a), such anomalies correspond to a poleward-shifted Pacific jet. At lag −4 days, the Rossby wave train is still present but new anomalies appear upstream in the western North Pacific with a ridge centered at 25°N and a trough at 50°N near 90°–120°W. Significantly more precipitation now appears in the subtropical regions south of Japan (Fig. 4c) associated with increased available moisture and northeastward oriented moist fluxes (Fig. 4d), which are not present during the preceding four days (Fig. 4b). Between lags −4 days and lag 0 day, the upper-level trough moves from Central Asia to western North Pacific where it interacts with the strong baroclinicity associated with the presence of land-sea thermal contrasts leading to surface cyclogenesis. The presence of a minimum 800 hPa geopotential anomaly downstream of the minimum 300 hPa geopotential anomaly in Fig. 4f reveals strong baroclinic interaction that has been reinforced by the presence of more moisture at the entrance of the storm-track region. This event closely resembles events of high warm conveyor belt activity in the western NPac^43^. At lag +4 days, the upper-level trough and its downstream ridge are reinforced (Fig. 4g) and the surface cyclone propagates downstream and poleward (black contour in Fig. 4h). In conclusion, two precursors of tropical origin favor PAC precipitation events. The first is a quasi-stationary Rossby wave train, triggered by suppressed convection in the Central Pacific. It reinforces the upper-level jet and baroclinicity in the PAC region prior to the synoptic event. The second is the larger amount of available moisture in the subtropical western NPac, which is picked up by the mid-latitude synoptic event during baroclinic growth.

**Fig. 5:**
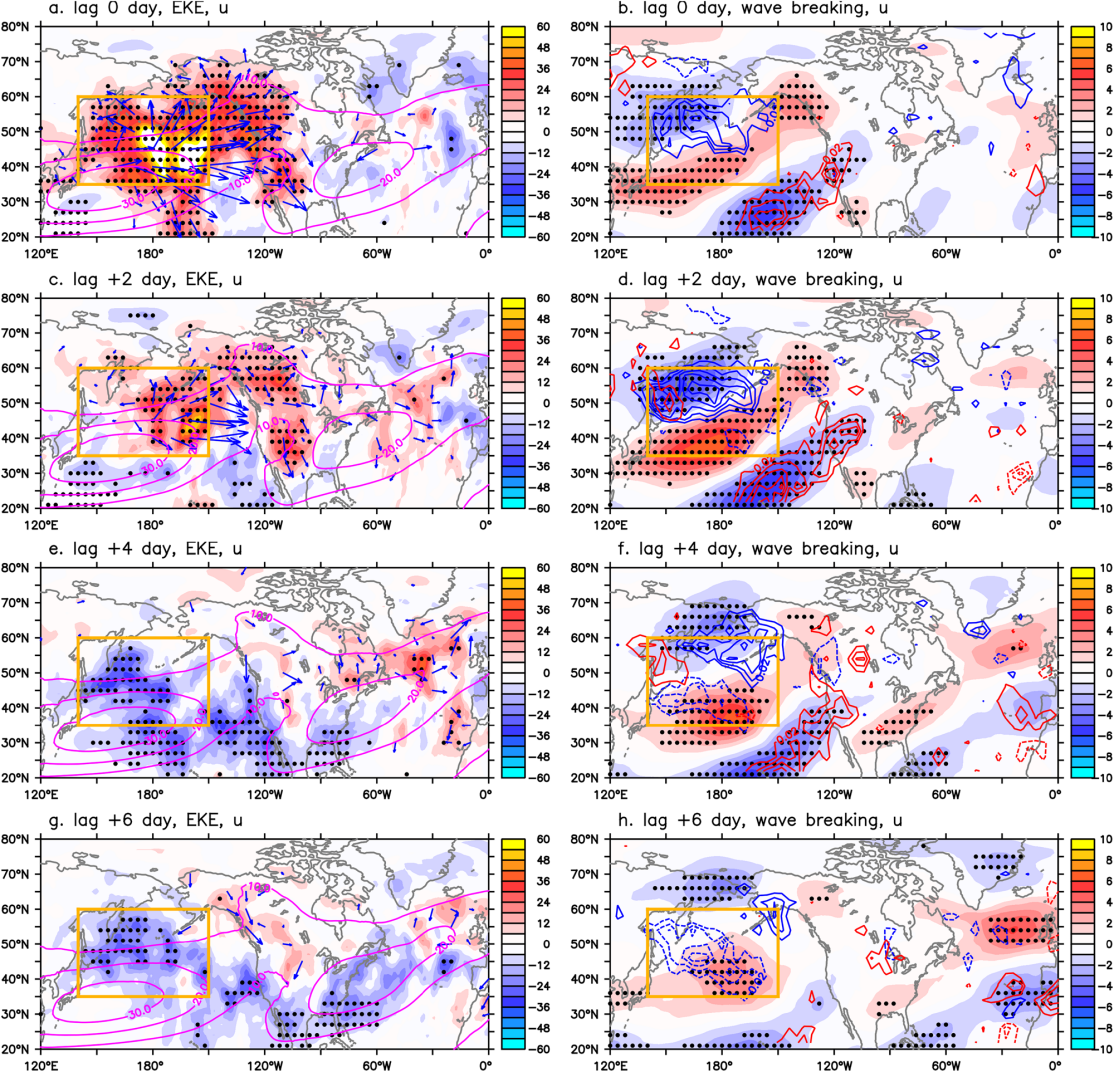
Time-lag anomalous composites of eddy kinetic energy (EKE), E-vectors, zonal wind and wave-breaking frequencies for intense precipitation events in PAC (Pacific region delimited by the orange box). (left column) eddy kinetic energy anomalies at 300 hPa (shadings; units: m^2^/s^2^), vertically averaged zonal wind between 300 and 800 hPa (magenta; contours: 10 m/s) and E-vectors at 300 hPa (blue arrows in regions where EKE anomalies are positive). (Right column) the vertically averaged zonal wind anomalies between 300 and 800 hPa (shadings; unit: m/s) and Rossby wave breaking frequency anomalies averaged over 300-K, 315-K, 320-K, 330-K and 350-K isentropic surfaces (red contours for anticyclonic wave breaking; blue contours for cyclonic wave breaking; positive and negative values are shown in solid and dashed contours; units: 0.01 day^−1^ for absolute values larger than 0.02 day^−1^). Each quantity is averaged over 3 days and centered at the indicated lag. Black dots indicate significance at the 5% level using a t-test for the eddy kinetic energy on the left column and the zonal wind on the right column. Time lags are **a**, **b** 0 day, **c**, **d** +2 days, **e**, **f** +4 day, **g**, **h** +6 day.

Figure 5 details the subsequent evolution of synoptic eddy activity and jet streams following such a prolonged precipitation event in the PAC region. During the event (lag 0 day), intense high-frequency eddy kinetic energy (EKE) is produced in the PAC region (Fig. 5a). It is associated with intense cyclone wave-breaking (CWB) in the western NPac over the PAC region where a surface cyclone is formed as confirmed by the poleward E-vectors^44^ at that location (Fig. 5a) and by the positive anomaly of CWB frequencies (Fig. 5b). Downstream of the PAC region, in the eastern NPac, anticyclonic wave-breaking occurs as seen by the more equatorward E-vectors (Fig. 5b) and the positive anomaly of AWB frequencies (Fig. 5d). CWB are known to accelerate the jet equatorward^45,46^. Here, it creates an acceleration of the jet on the southern part of the PAC region and a deceleration on its northern side. Since the box is located north of the jet axis, such an anomalous pattern accelerates the Pacific jet north of its axis. On the other hand, AWB classically accelerate the jet poleward and thus leads to an acceleration of the jet in the eastern NPac that reinforces the large-scale ridge. After the event, a large part of EKE propagates downstream, reaching high significant positive anomalies over North America at lag +2 days (Fig. 5c) and subsequently significant positive EKE anomalies emerge over the eastern NAtl at lag +4 days (Fig. 5e). At lags +4 and +6 days, the Atlantic jet is strongly accelerated along its axis on its eastern side, consistent with more CWB and AWB on its flanks (Fig. 5f, h). Such a jet acceleration in the eastern NAtl resembles the positive NAO phase (see the dipolar geopotential anomaly in Fig. 4g) and the intense downstream propagation of EKE from the NPac to the NAtl is similar to the positive Northern Annular Mode phase^47,48^.

## Discussion

This study provides a dynamical explanation for the poleward-shifted NPac jet and accelerated NAtl jet observed in reanalysis data during the last four decades. Several major findings can be listed from our study. Precipitation and diabatic heating increase in the storm-track regions north of their climatological regions in both NPac and NAtl. The increased mid-latitude precipitation in NPac is favored by La-Niña and MJO-phases 4 to 6 tropical precipitation patterns characterized by more precipitation over the Maritime Continent. The one in NAtl is connected to increased precipitation in the ITCZ region of Central Pacific and more to El-Nino events. The increased mid-latitude precipitation in NPac is closely related to jet stream trends in both NPac and NAtl regions whereas the increased mid-latitude precipitation in NAtl is only partly related to jet stream trends even in the NAtl. There are twice as many intense sustained precipitation synoptic events in the NPac (PAC region) during the recent period (2002–2024) as during the old period (1979–2001). Those sustained events are associated with a poleward-shifted jet stream in the NPac followed by an accelerated NAtl jet a few days later. The events are favored by La-Niña like tropical convection anomalies via two effects. One is the excitation of a Rossby wave train in the Central Pacific leading to an intensification of the Pacific jet on the poleward flank of its climatological position. This creates a favorable baroclinic environment for subsequent cyclogenesis events, which are typically initiated by baroclinic interaction with upper-level disturbances entering the NPac from upstream. The other is more moisture in the subtropical western NPac that provides latent energy for upcoming surface cyclogenesis. Hence, these two effects favor the triggering of intense cyclogenesis events on the poleward flank of the Pacific climatological jet axis. Such events lead to large dispersion of eddy kinetic energy downstream into the NAtl region where the jet is accelerated.

The combined effects of quasi-stationary and synoptic eddies underlined in the present paper are similar to those described during positive Northern Annular Mode events^48^ or those explaining the impact of MJO or ENSO onto the North Atlantic Oscillation (NAO)^47,49,50^.

There is a priori no direct link between the long list of mechanisms described in the literature to interpret the poleward-shifted jet streams in global warming scenarios^18^ and the one presented here to interpret the detected jet stream trends in the reanalyzes. One big difference is that the former mechanisms mainly rely on a zonal-mean picture of the thermodynamical changes under global warming while the present mechanism is closely related to zonal asymmetries in tropical precipitation trends. The present study shows evidence of the link between precipitation trends in the tropics and increased synoptic-scale events in the mid-latitude North Pacific. But circumstances under which such a link is valid need to be further investigated and the proposed mechanism needs to be tested in different ways, not only using reanalysis.

Under global warming, we expect an increase in precipitation in the storm-track regions but the key question would be to analyse where such increased precipitation occurs in historical and future climate simulations and how it depends on zonal asymmetries in tropical precipitation. Concerning historical simulations and their inability to reproduce observed jet stream trends there are two underlying questions, one is why coupled models are unable to reproduce La-Niña like SST trends, the other is why atmospheric-only simulations forced with historical SSTs are also unable to reproduce the observed trends, despite significant improvements relative to coupled models^22,23^.

## Methods

### Data

The main paper relies on daily and monthly outputs on 1^° ^× 1° resolution of ERA5 reanalysis^51^. The total precipitation is the sum of convective and large-scale precipitation associated with the convection and large-scale clouds schemes of the IFS model, respectively. As mentioned in ref. ^51^, radar and gauge precipitation data are only assimilated over the US and there is no assimilation of precipitation in other regions. In the supplement information, two other reanalysis datasets are used, the Japanese 55-year^[JRA-55; 52^, the Modern-Era Retrospective Analysis for Research and Applications^[MERRA-2; 53^ and three observations-derived (satellite and/or gauge) datasets, the Global Precipitation Climatology Product^[GPCP; 54^, the NASA’s Integrated Multi-satellitE Retrievals for GPM^[IMERG; 55^, the Climate Prediction Center morphing method^[CMORPH; 56^ and the Multi-Source Weighted-Ensemble Precipitation^[MSWEP V2.8; 57^.

### Statistical significance

In Fig. 1, the trends are estimated with least squares linear regressions and their statistical significance is based on the Student’s test of ref. ^58^ with the computation of an effective sample size. The total number of years is *n*_*t*_ = 46 for the 1979–2024 period but a correction is applied to take into account the dependency of the datasets with time. A one-year lag autocorrelation *r*_1_ is computed to get an effective sample size *n*_*e*_ = *n*_*t*_(1 − *r*_1_)/(1 + *r*_1_) and the test is applied for *n*_*e*_ − 2 degrees of freedom. Additionally, we have applied another estimator of the trend and the non parametric Mann-Kendall test for the trend and the results do not change much (compare Figs. 1 and S9). The statistical test of the composites shown in Figs. 4 and 5 is a Student t test with the computation of an effective sample size where the autocorrelation is computed with a lag of 24 days which is the averaged time difference between two consecutive precipitation events.

### Selection of precipitation events

To select the sustained precipitation events in the PAC and ATL regions, we low-pass filter the daily precipitation (periods greater than 10 days) and then consider all the days for which the averaged low-frequency precipitation in each of these regions is beyond 1.5 standard deviation (red parts of the curves in Fig. S5) and consider the lag 0 of the event when the averaged low-frequency precipitation is greater than 2 days before and 2 days after (red circles in Fig. S5). If we do not apply a low-pass filter and work with the daily precipitation field, the differences between the recent period (2001–2022) and the old period (1979–2000) are less.

### ENSO and MJO indexes

The ENSO index is the area-averaged monthly anomalies over the Niño 3.4 region (5°N-5°S)(170°-120°W) using a climatology of 1981–2010 and the Met Office Hadley Centre’s sea ice and SST data set^[HadISST1,59^. The MJO index is based on the Real-time Multivariate MJO index^60^ and downloaded from https://www.bom.gov.au/climate/mjo/.

### Analysis tools

The low-pass filter is a nine-point Welch window with a 10-day cutoff period^48^. High-frequency kinetic energy is then computed $\,{\mbox{EKE}}\,=({u}^{{\prime} 2}+{v}^{{\prime} 2})/2$ as well as the so-called E-vector ${{\bf{E}}}=\left(({v}^{{\prime} 2}-{u}^{{\prime} 2})/2;-{u}^{{\prime} }{v}^{{\prime} }\right)$^44^. The wave-breaking detection method is based on the overturning of the daily potential vorticity circumpolar contours on four isentropic levels, 300 K, 315 K, 320 K, 330 K and 350 K^61,62^. The circumpolar contours are globally west-to-east oriented and each segment of the contour locally oriented from east to west is identified as belonging to a wave-breaking segment. If the segment is northeast to southwest oriented it is identified as an anticyclonic wave-breaking (AWB) segment. If it has a southeast-northwest orientation, it is identified as a cyclonic wave-breaking (CWB) segment. Frequencies of occurrence of AWB and CWB segments are then positioned onto the 1° × 1° grid as the other variables.

## Supplementary information

Supplementary Information

Transparent Peer Review file

## Data Availability

The ERA5 reanalysis data used in this study are available in the Copernicus Climate Data Store: https://cds.climate.copernicus.eu^63^.
