# Supplementary material for: Tropical origins of the recent trends in Northern Hemisphere wintertime jet streams
Source: Nat Commun. 2026 Jun 29;17:8115. doi: 10.1038/s41467-026-74980-3 (PMC13458359; doi:10.1038/s41467-026-74980-3)
Supplement: Supplementary file 1 [file 41467_2026_74980_MOESM1_ESM.pdf]

Supporting Information for "Tropical origins of the recent trends in Northern Hemisphere wintertime jet streams"

Gwendal Rivière[1], Sébastien Fromang[2], Sebastian Schemm[3*]

[1]LMD/IPSL, CNRS, Ecole Normale Supérieure, Université PSL, Sorbonne Université, Ecole Polytechnique, IP Paris, Paris, France.
[2]LSCE/IPSL, CEA, CNRS, UVSQ, Université Paris-Saclay, Gif-sur-Yvette, France.
[3*]Department of Applied Mathematics and Theoretical Physics, University of Cambridge, Wilberforce Road, Cambridge, CB3 0WA, UK.

Summary of the supplement information The supporting information contains 8 figures. Figure S1 is the same as Figure 1b of the paper but with EKE trends computed at two distinct levels 300 hPa and 800 hPa. Figures S2 to S4 show various precipitation trends computed with various datasets. Figure S5 illustrates how sustained precipitation events in the PAC region are selected by showing the 2021-2022 winter as an example. Figures S5, S6 and S7 are the same as Figures 3, 4 and 5 of the paper but computed for the sustained precipitation events occurring in the ATL region. Figure S9 is the same as Fig. 1 of the paper but using a different trend estimator and a different statistical test.

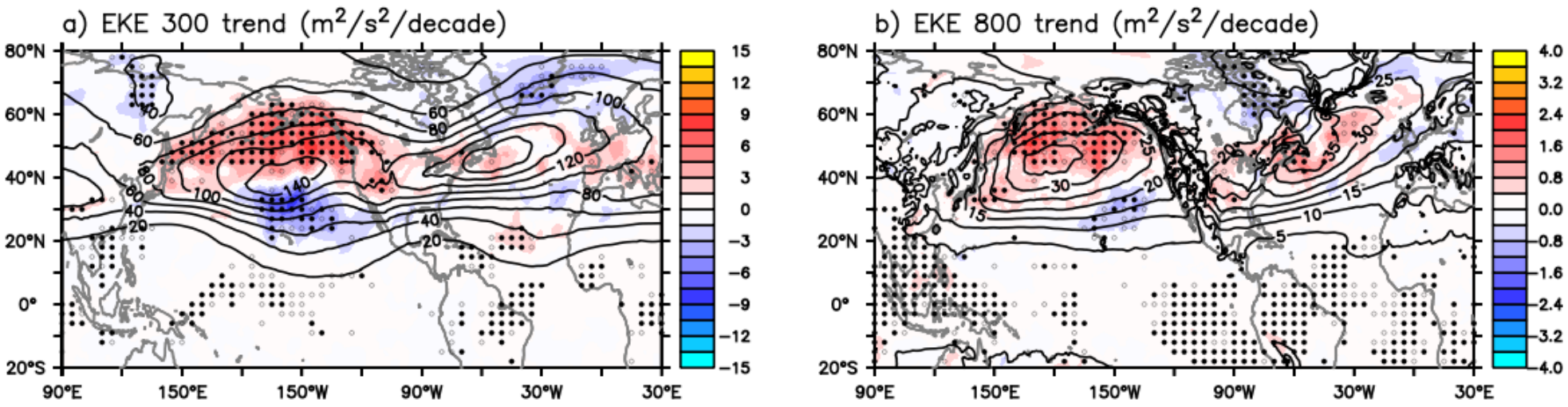

Fig. S1 Least squares linear regression trends during DJF from 1979 to 2024 in Eddy Kinetic Energy (EKE; shadings; units: $m^2\ s^{-2}\ decade^{-1}$) at a) 300 hPa and b) 800 hPa. Contours show the climatology. Data: ERA5 reanalysis. Black dots and open circles indicate significance at the 5% and 10% levels respectively using a two-sided t-test.

Supporting notes

When considering the full period of interest (1979–2024), four products exhibit significant increase in precipitation in the storm-track regions north of their climatology (ERA5, JRA-55, MERRA-2, MSWEP) while GPCP does not show any significant increase in those regions (Fig. S2). One could hypothesize the reanalysis-derived products have biases and might be wrong, but satellite-derived products over the oceans do not agree either during the 2000–2024 period (Fig. S3 and S4). IMERG shows a much more rapid increase than in reanalysis, CMORPH a similar increase, while GPCP again does not indicate any positive trend in the storm-track regions.

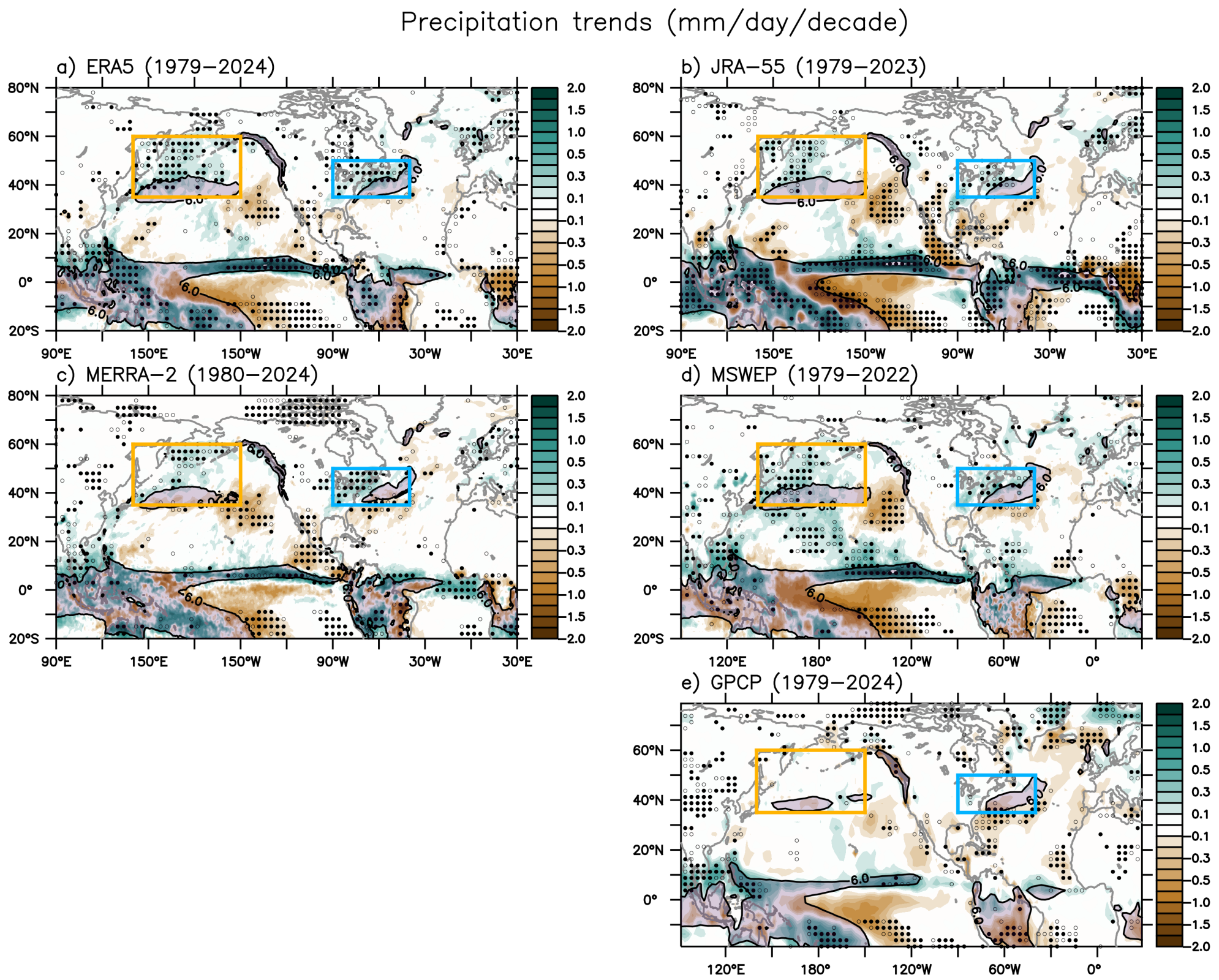

Fig. S2 DJF least squares linear regression trends during the last four decades in total precipitation (shadings; units mm day^{-1} decade^{-1}) and climatology (black contour and shaded for values greater than 6 mm day^{-1}): a) ERA5 reanalysis, b) JRA-55 reanalysis, c) MERRA-2 reanalysis, d) MSWEP multi-source product, e) GPCP based on satellite and Gauge observations. Black dots and open circles indicate significance at the 5% and 10% levels respectively using a two-sided t-test.

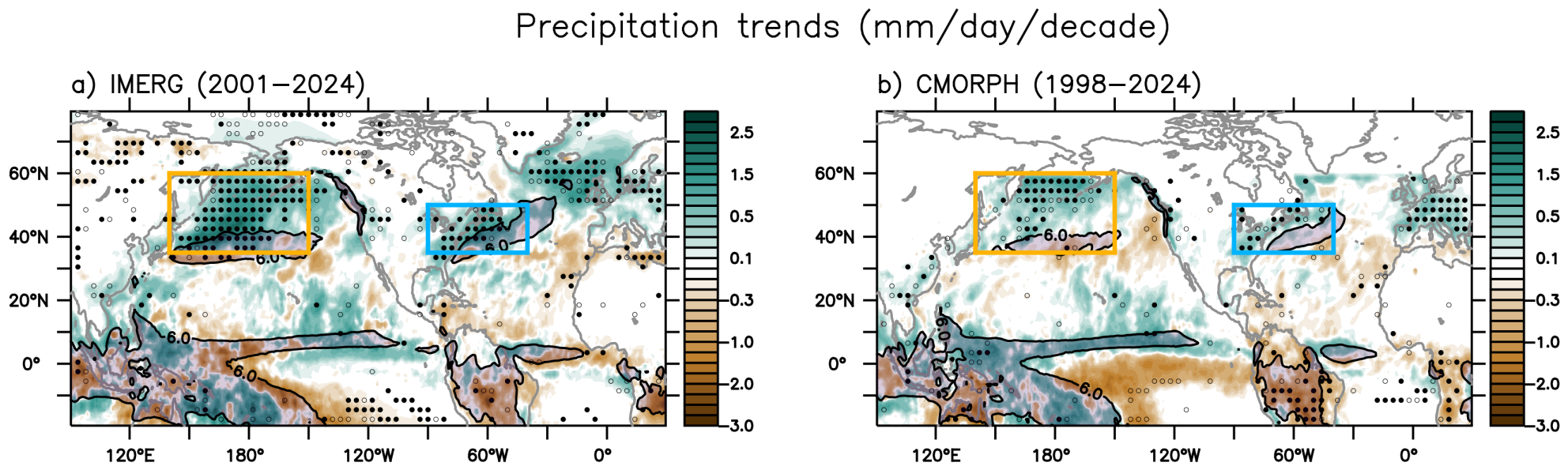

Fig. S3 DJF linear trends during the last two decades in total precipitation (shadings; units mm day^{-1} decade^{-1}) and climatology (black contour and shaded for values greater than 6 mm day^{-1}): a) IMERG satellite product, b) CMORPH satellite product. Black dots and open circles indicate significance at the 5% and 10% levels respectively using a two-sided t-test.

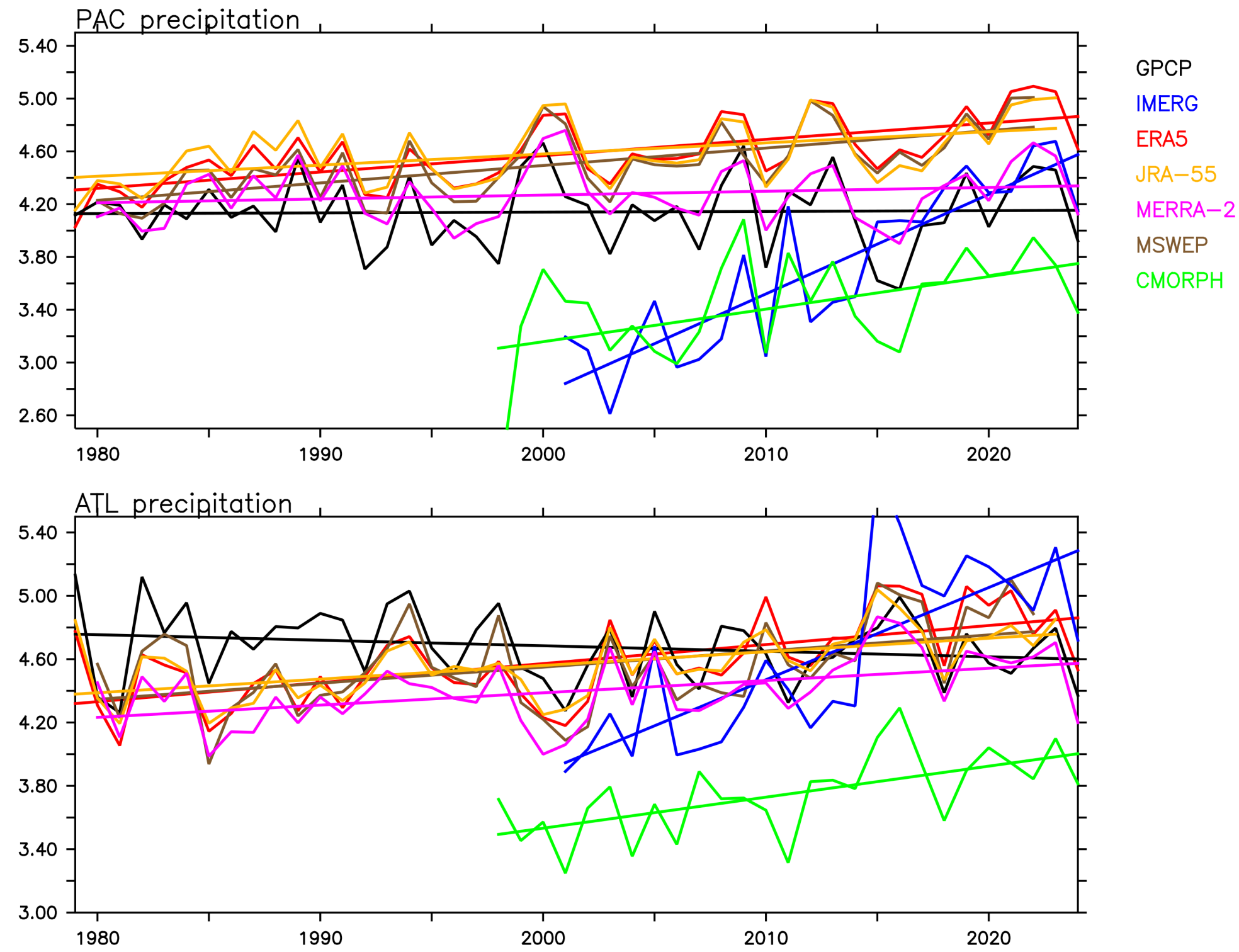

Fig. S4 Time evolution of DJF total precipitation for 7 products and their linear regressions: 3 reanalysis (ERA5, JRA-55, MERRA-2), one multi-source product (MSWEP) and other observational datasets based on satellite and/or gauge measurements (GPCP, IMERG, CMORPH).

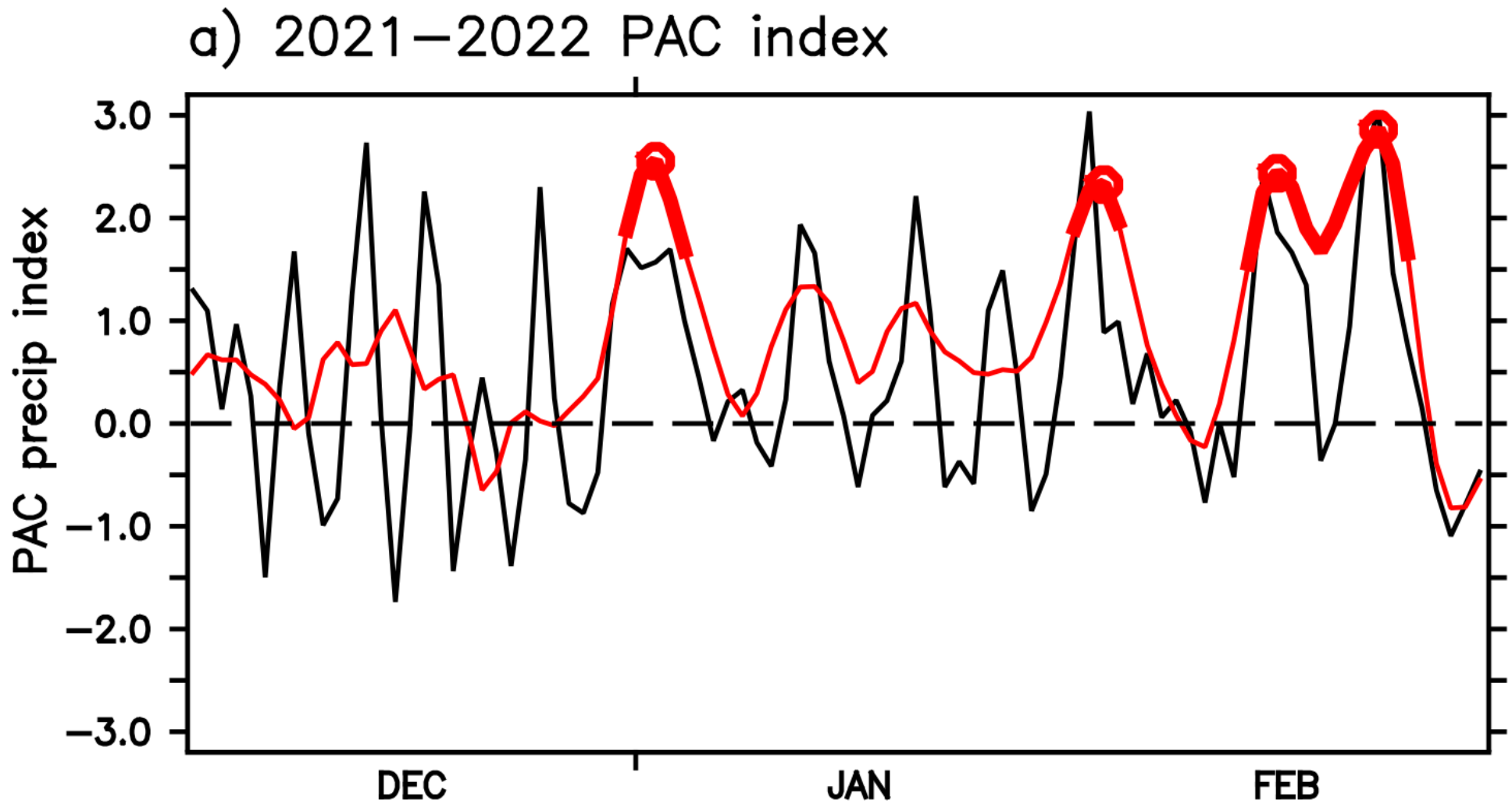

Fig. S5 Example of selection of sustained precipitation events in the PAC region (orange box in Fig. S2 and S3). The time evolution of total precipitation averaged over the PAC region (black contours) and its low-frequency component (red contours) are shown during winter 2021-2022. The thick red lines correspond to the values beyond 1.5 standard deviation and the red circles the maxima over a 5 days period.

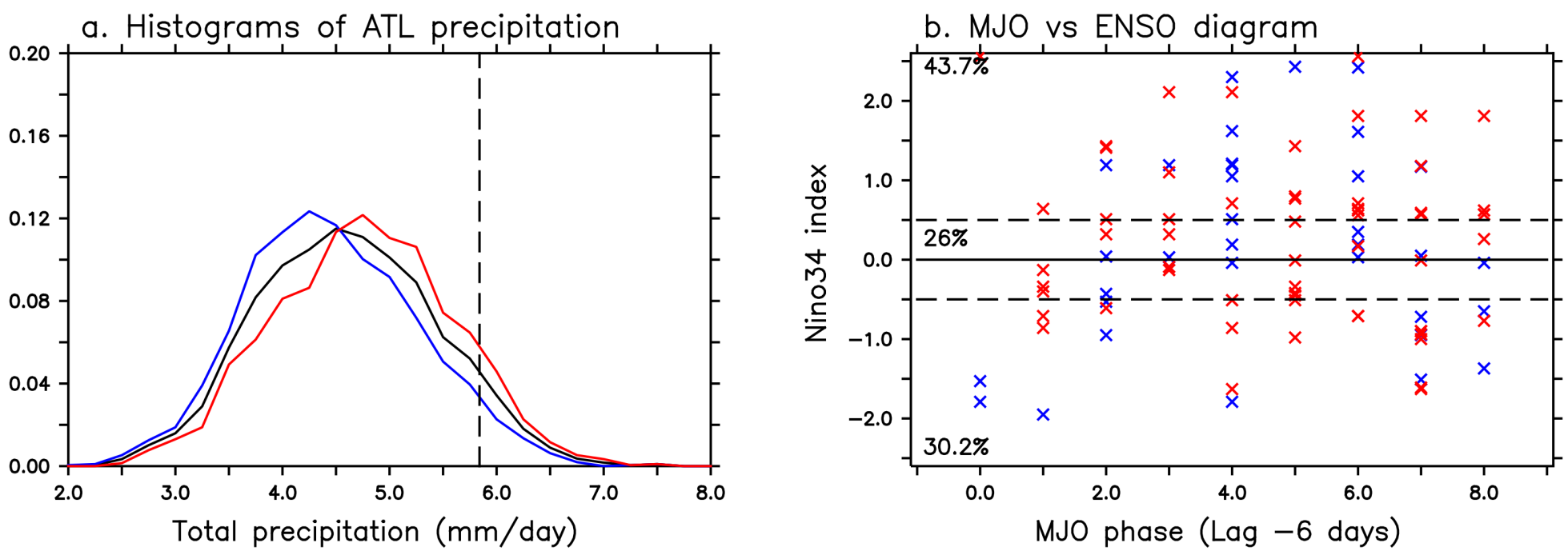

Fig. S6 Selection of sustained heavy precipitation events in the North Atlantic and links with MJO and ENSO. Same as in Fig. 3 but for the ATL region (see blue box in Fig. S2).

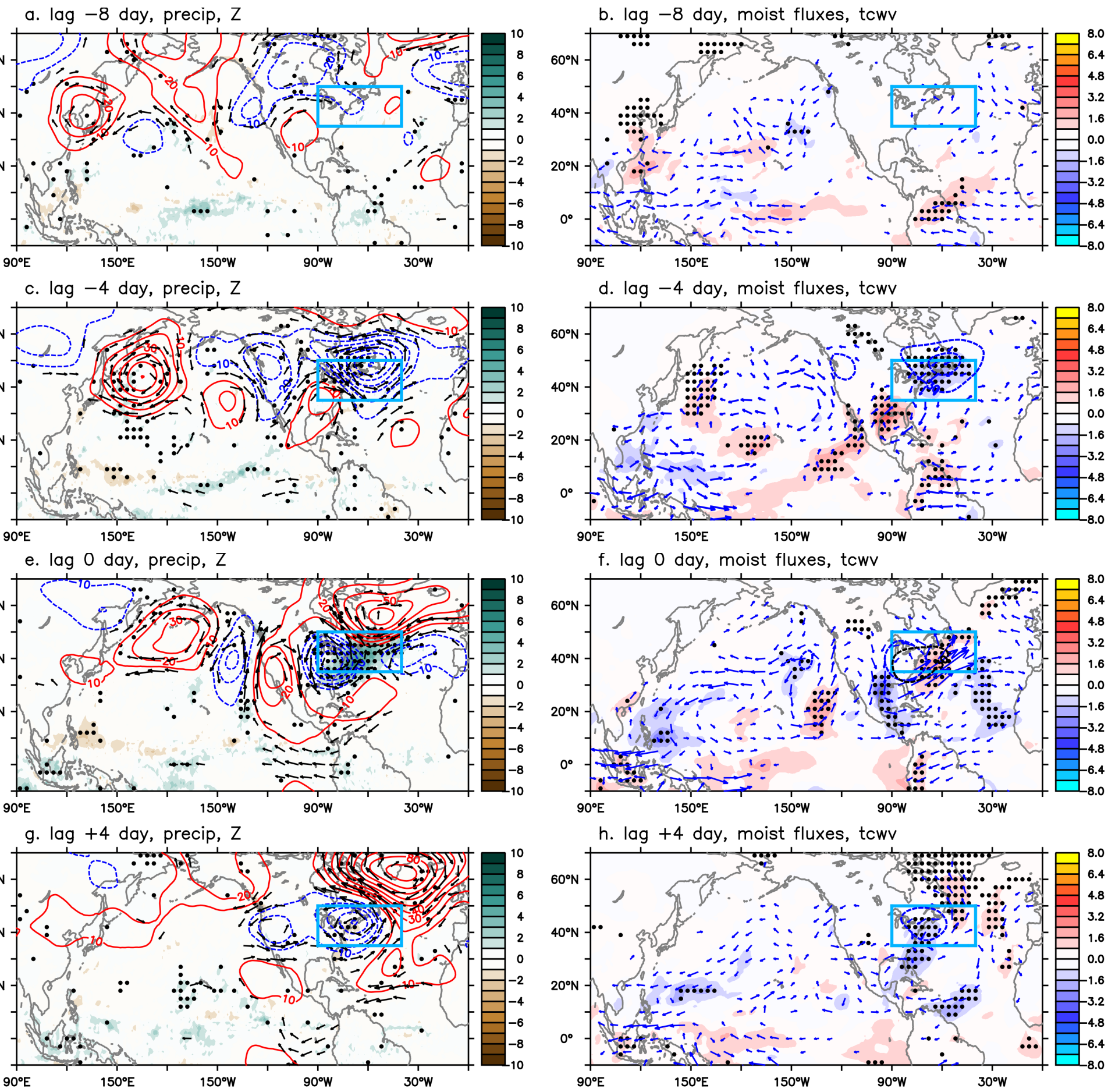

Fig. S7 Time-lag anomalous composites of geopotential, precipitation and moisture for intense precipitation events in the ATL region (blue box). Same as Fig. 4 but for the ATL region.

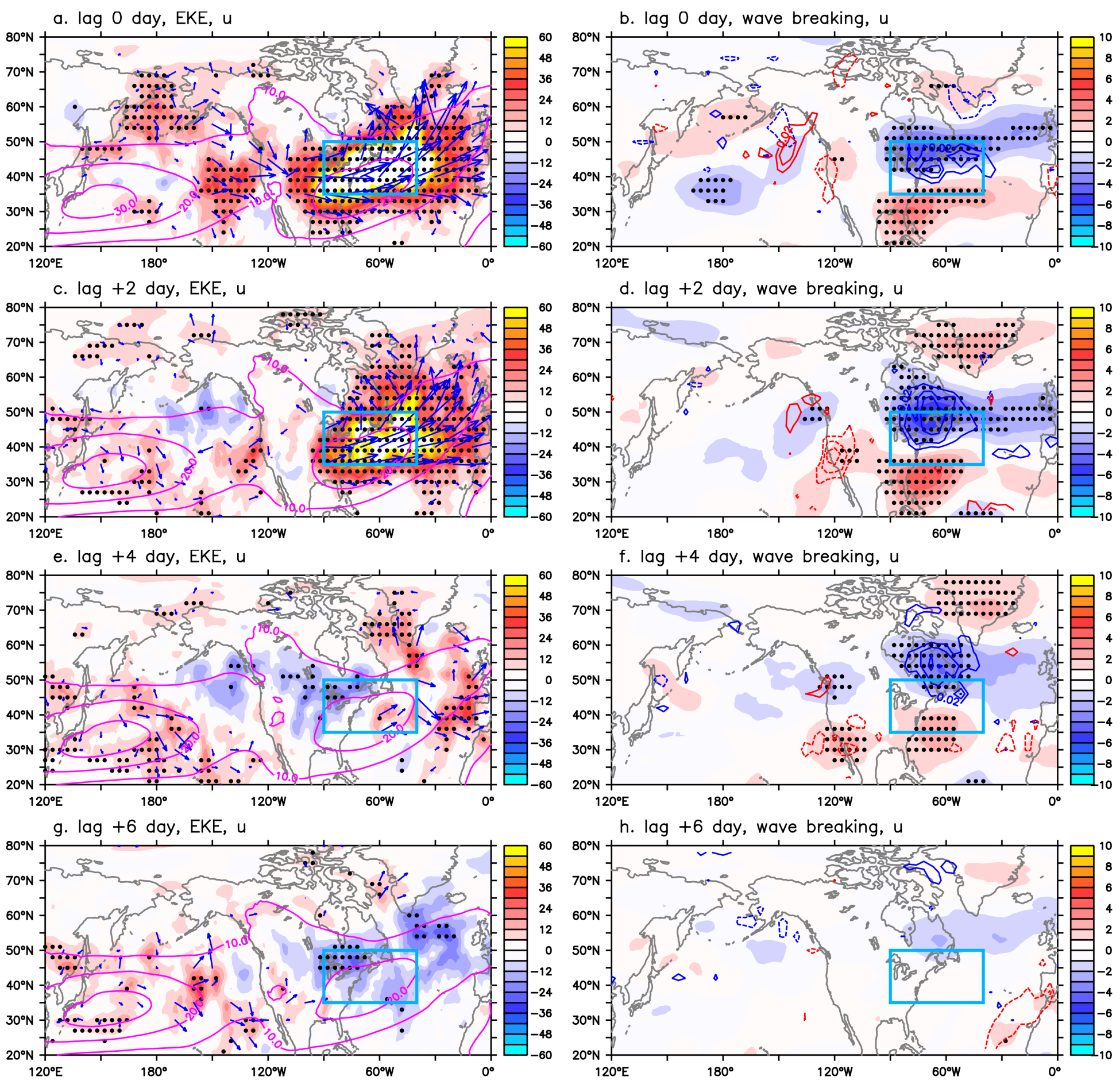

Fig. S8 Time-lag anomalous composites of eddy kinetic energy, E-vectors, zonal wind and wave-breaking frequencies for intense precipitation events in the ATL region (blue box). Same as Fig. 5 but for the ATL region.

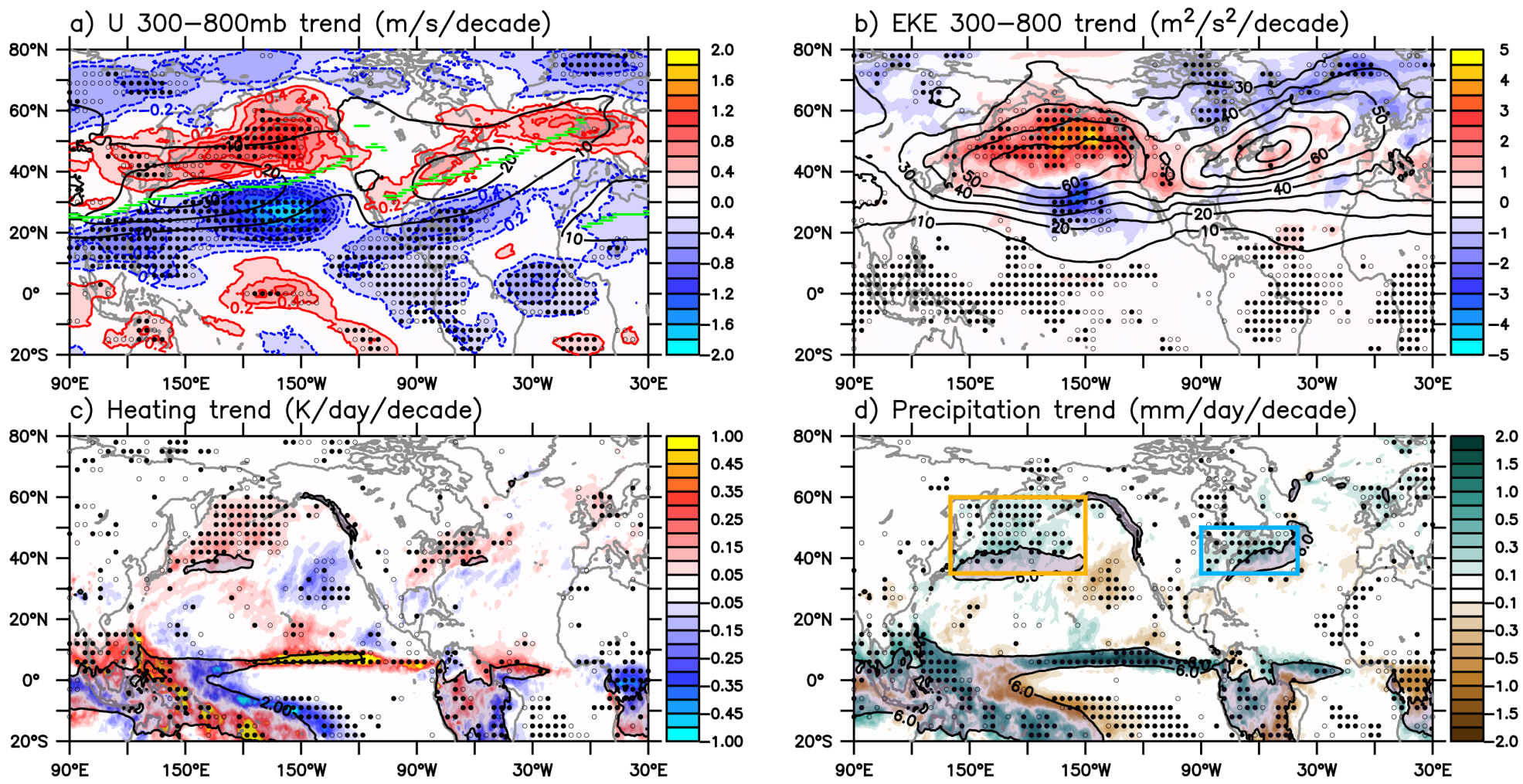

Fig. S9 Same as in Figure 1 of the main paper but using the Theil-Sen's slope estimator and the non parametric Mann-Kendall test for monotonic trend.
