# Supplementary material for: Tropical origins of the recent trends in Northern Hemisphere wintertime jet streams
Source: Nat Commun. 2026 Jun 29;17:8115. doi: 10.1038/s41467-026-74980-3 (PMC13458359; doi:10.1038/s41467-026-74980-3)
Supplement: Supplementary file 2 [file 41467_2026_74980_MOESM2_ESM.pdf]

nature portfolio

Peer Review File

Tropical origins of the recent trends in Northern Hemisphere wintertime jet streams

Corresponding Author: Professor Sebastian Schemm

This file contains all reviewer reports in order by version, followed by all author rebuttals in order by version.

Version 0:

Reviewer comments:

Reviewer #1

(Remarks to the Author)
In this work Rivière et al. analyse observed trends in the Northern Hemispheric Jet Stream focusing on the Atlantic and particularly the Pacific basin. Since coupled climate models do not reproduce this trend well, the authors opt for analysing reanalysis data over the sattelite period only to explain the trend. The authors show a relationship between trends in the precipitation in the jet entry region and midlatitude zonal winds. They further show a link between ENSO and MJO and heavy preciptation in the North Pacific region. In a final step the authors propose, grounded in data analysis, a mechanism how tropical precipitation anomalies trigger quasi-stationary Rossby waves that propagate into the mid-latitude and drive precipiation and wind anomalies there. Given the trend in ENSO, the implicit conclusion is that the trends in precipitation and winds in the mid-latitude are tied via this mechanism to the tropical trends.

This study tackles an open question about the emergent trends in the Northern Hemispheric circulation and precipitation and is therefore timely and relevant. The manuscript is very well written and the figures are of high quality. While I find the presented analysis logical and well thought-out, I have some concerns about the statistical significance testing, which I believe was not done carefully enough. Given that the authors opted to only consider reanalysis data for this study (a choice I follow, but think that model (experiment)s could still provide insight), and the argument therefore rests on this analysis only, the statistical testing should be done with extra care. Addressing these concerns might reduce the significance of the findings and require put caveats on the conclusions For this reason I am recommending major corrections, but am confident that these can be addressed.

Major comments

As stated above, I am concerned about the statistical significance testing and will give more detail in the following.

As a minor comment upfront, why is the significance level varied between the different analyses? It takes values of 90%,95%,98% and 99%?

My main concern is however the use of a simple t-test for most significance testing. The t-test assumes data to be independent, which is not the case when considering timeseries. In this case either a different test has to be chosen, that respects the autocorrelation in the data or some other preprocessing like for example pre-whitening should be done. As far as I can see, this applies to the results in Fig.1, Chapter 4 paragraph 1,

Additionally, one should control for multiple testing, when testing every grid points independently as in Fig.1, Fig.4, Fig.5. With a significance level of 95%, you would expect 5% of the points to be significant, just by chance. when not controlling for multiple testing. One way to control for this is for example described by Wilks 2016 (DOI:10.1175/BAMS-D-15-00267.1).

I expect some changes in the significance of some results, which would mean some rephrasing or re-interpretation of the results. I am not trying to be annoying or nitpicky, and I know that many studies in our fields are not as careful, but I do think it is very important, to avoid overstating confidence in results.

Finally about Fig5: Was the significance of the winds/geopotential tested? This seems important, as the argument rests on

the Rossby wave propagating poleward.

Minor comments

Introduction
p.2 “This contradicts the sensitivity…”, I find the word ‘contradicts' too strong here. There are many factors that will ultimately determine the jet streams response as there are a multitude of (possible) mechanisms and drivers, so even if the models simulate a stronger tropical warming, it does not mean that the overall response has to be a stronger shift. While I agree with the sentiment of the statement, I would soften it.

p.2 “… obviously not for the same mechanistic reason…”, Again I find this too strongly worded and not necessarily backed up by the references. Simpson et al. 2025 states in regard of the zonal mean jet shift in relation to tropical warming issue ' but that they may not have the correct relationship between upper tropospheric warming and this poleward shift, so this may be for the wrong reasons.’

Chapter 2
Is there a reason why data from 2023 and 2024 was excluded?

Chapter 3
After reading the whole manuscript, I am a bit confused about the wording in the interpretation of the regression analysis here. Before reading all the way through, I had the impression that you were arguing that trends in the precipitation are driving the trends in the zonal winds due to phrases like “To conclude, the NPac jet trend is directly linked to NPac precipitation trend” or ‘…contribution of the PAC and ATL precipitation to the zonal wind trends can be estimated by …’. I was already prepared to point out that this can not be concluded from the regression analysis, since the influence might be bi-directional or due to a common driver of both of them. After reading the whole manuscript I understand that you are arguing, that indeed trends in the tropics are the ultimate drivers of these trends (at least in parts). Maybe it is just me, but I would rephrase some parts of this chapter to make sure that it is not misunderstood to be seen as causal.

Fig.1: I would be consistent between subfigures regarding if shading or contours are used for climatology and trends respectively

Technical

compage -> compare

Line numbers would be helpful

Reviewer #2

(Remarks to the Author)
The paper is clearly written and presents a plausible mechanism that could explain observed recent trends in jet streams whose cause is uncertain. Explaining these trends is a significant topic in the climate/dynamics. The results are not conclusive in the sense that the authors do not state that the mechanism presented is responsible for the observed trends, merely that it is a candidate mechanism for which they present some evidence. This is to be expected given the uncertainties involved.

I have one major methodological question:

The Ipac and Iatl indices are selected to be in regions where there are large trends. These are then regressed against the zonal jets which also have trends. Finally the regression coefficients and trends from the Ipac and Iatl indices are used to calculate the "trend congruent with" the zonal winds. A question that immediately arises is, how much of regression coefficient between the winds and rainfall indices is simply due to the fact that both have trends - to address this a suggestions would be to calculate the regression coefficients again after detrending both datasets.

Minor Comments:
- discussion of rainfall datasets - suggest an additional sentence or so discussing what rainfall observations are assimilated into ERA5 and any implications for the two specific regions selected.

- Comparison of Fig 1a and Fig 2a - this is a bit awkward as they are not in the same figure. Suggest strengthening the assertion that they are similar with a pattern correlation value.

- Fig 4, 5: Significance at 2% is indicated by black dots - since there are multiple fields show in each panel it is unclear which is significant. If not all fields are significant how does this effect the interpretation of results.

Reviewer #3

(Remarks to the Author)
Review of Rivière et al., “Tropical origins of the recent trends in Northern Hemisphere wintertime jet stream”

Rivière et al. present a well-conceived study that provides a novel and compelling explanation for the observed trends in Northern Hemisphere wintertime jet streams over recent decades. As the authors note, most previously proposed mechanisms explaining the jet response to global warming are framed in zonal-mean terms, where tropical upper-tropospheric warming or Arctic amplification are considered the primary drivers of change. In contrast, the present manuscript proposes a mechanism in which asymmetric trends in tropical Pacific precipitation force a poleward displacement of the North Pacific (NPAC) jet and an acceleration of the North Atlantic (NATL) jet.
Overall, the methods are sound and the proposed mechanism plausible. I find the manuscript worthy of publication. However, I would like the authors to clarify a few points before publication.

(1) The main conclusion of the manuscript is that asymmetric changes in tropical precipitation are related to the historical jet trends. The proposed causal chain is that tropical precipitation anomalies force a Rossby wave response that accelerates the poleward flank of the jet. In addition, the authors argue that these anomalies induce enhanced precipitation, increased cyclogenesis, and wave breaking at the western North Pacific boundary, which further reinforces the poleward displacement of the jet.

However, in Figure 1 the authors quantify how midlatitude precipitation anomalies (orange boxes) contribute to the jet trends rather than directly quantifying the contribution of tropical precipitation anomalies, which are central to the proposed mechanism. The authors could consider applying a lagged version of their regression framework using tropical precipitation as a predictor. Such an analysis would provide a more direct link between tropical anomalies and jet trends and would strengthen the evidence supporting the mechanism described.

(2) In the introduction the author’s state:
“This contradicts the sensitivity of the jet poleward shift amplitude to the upper-tropospheric temperature gradient described above [18, 19]”.
I am not sure contradicts is the right terminology here since some papers have shown that the jet response to tropical warming not only depends on its magnitude but it strongly depends on the structure of the warming. See e.g., : Wang, S., Gerber, E. P., & Polvani, L. M. (2012). Abrupt circulation responses to tropical upper-tropospheric warming in a relatively simple stratosphere-resolving AGCM. Journal of Climate, 25(12), 4097–4115.
https://journals.ametsoc.org/view/journals/clim/25/12/jcli-d-11-00166.1.xml

(3) Directly after the sentence I commented in point (2) the authors state:
“Therefore although the models are able to qualitatively reproduce the observed jet streams trends…”.
This statement appears somewhat overconfident. Several studies have shown that model agreement with observed jet trends depends strongly on the region, season, and wind level considered. For instance, model performance varies substantially between the North Pacific and North Atlantic basins and across different pressure levels. The authors may wish to moderate this statement or acknowledge this variability in model performance (see, e.g., Ossó et al. for a review).

Version 1:

Reviewer comments:

Reviewer #1

(Remarks to the Author)
This is my second review of the manuscript and for the sake of discussion, I am waiving my anonymity.

I thank the authors for redoing the analysis so thoroughly. I am a bit surprised that the FDR approach reduced the significance as much as it did. Did the authors follow Wilks’s suggestion to use an FDR significance level that is doubled to the base significance level to account for spatial correlation?

I am myself not fully sure what is the correct way to deal with significance testing on trends of spatially and temporally resolved fields, but to my understanding the two tests can be done consecutively, as one deals with the temporal correlation and the other with the problem of multiple testing. I have applied a similar approach, namely a pre-whitening and an FDR correction consecutively before (Fig. 5 or S1; https://doi.org/10.1038/s43017-025-00749-9). And I do believe that both things have to be accounted for.

However, I am not going to push the point further, since I know it is unconventional in the field and I further think the paper is well designed and written and is very interesting. But, if the authors are interested in discussing further, I am very keen to do so, since I have thought (and searched in the literature) about how to do testing on trends of spatially and temporally resolved fields several times now. This seems like it should be a standard tool in the field, shouldn’t it?

I have no further comments on the manuscript and can recommend it for publication.

Philipp Breul

Reviewer #2

(Remarks to the Author)
The authors have satisfactorially addressed the concerns raised in review. Overall the paper is clearly written and presents a significant contribution to an important but uncertain topic in climate science. The methods are sound and reproducible, and the conclusion justified.

Reviewer #3

(Remarks to the Author)

The authors have carefully addressed my concerns. However,I do still have some reservations regarding the proposed mechanism. In particular, the regression maps with tropical precipitation are not conclusive and the causal direction of the relationships is not directly addressed by this study.

That said, although the mechanism is not definitely proven,the authors show enough evidence to considered it plausible and opens an interesting avenue for further investigation. Moreover, the authors have appropriately moderate the tone of their claims.

Overall, I find the article worthy of publication by Nature Communications.

Open Access This Peer Review File is licensed under a Creative Commons Attribution 4.0 International License, which permits use, sharing, adaptation, distribution and reproduction in any medium or format, as long as you give appropriate credit to the original author(s) and the source, provide a link to the Creative Commons license, and indicate if changes were made.
In cases where reviewers are anonymous, credit should be given to 'Anonymous Referee' and the source.
The images or other third party material in this Peer Review File are included in the article’s Creative Commons license, unless indicated otherwise in a credit line to the material. If material is not included in the article’s Creative Commons license and your intended use is not permitted by statutory regulation or exceeds the permitted use, you will need to obtain permission directly from the copyright holder.
To view a copy of this license, visit https://creativecommons.org/licenses/by/4.0/

Authors' response

Reviewers' comments are in black, authors' answers in blue

We would like to thank the three reviewers for their constructive comments and suggestions

Reviewer 1

In this work Rivière et al. analyse observed trends in the Northern Hemispheric Jet Stream focusing on the Atlantic and particularly the Pacific basin. Since coupled climate models do not reproduce this trend well, the authors opt for analysing reanalysis data over the sattelite period only to explain the trend. The authors show a relationship between trends in the precipitation in the jet entry region and midlatitude zonal winds. They further show a link between ENSO and MJO and heavy preciptation in the North Pacific region. In a final step the authors propose, grounded in data analysis, a mechanism how tropical precipitation anomalies trigger quasi-stationary Rossby waves that propagate into the mid-latitude and drive precipiation and wind anomalies there. Given the trend in ENSO, the implicit conclusion is that the trends in precipitation and winds in the mid-latitude are tied via this mechanism to the tropical trends.

This study tackles an open question about the emergent trends in the Northern Hemispheric circulation and precipitation and is therefore timely and relevant. The manuscript is very well written and the figures are of high quality. While I find the presented analysis logical and well thought-out, I have some concerns about the statistical significance testing, which I believe was not done carefully enough. Given that the authors opted to only consider reanalysis data for this study (a choice I follow, but think that model (experiment)s could still provide insight), and the argument therefore rests on this analysis only, the statistical testing should be done with extra care. Addressing these concerns might reduce the significance of the findings and require put caveats on the conclusions For this reason I am recommending major corrections, but am confident that these can be addressed.

Major comments

As stated above, I am concerned about the statistical significance testing and will give more detail in the following.

As a minor comment upfront, why is the significance level varied between the different analyses? It takes values of 90%, 95%, 98% and 99%?

The choice of several significant levels was done for aesthetic reasons and to avoid overloading some figures. In the revised paper, all figures systematically show the 95% level and additionally the 90% level in Fig.1.

My main concern is however the use of a simple t-test for most significance testing. The t-test assumes data to be independent, which is not the case when considering timeseries. In this case either a different test has to be chosen, that respects the autocorrelation in the data or some other preprocessing like for example pre-whitening should be done. As far as I can see, this applies to the results in Fig.1, Chapter 4 paragraph 1,

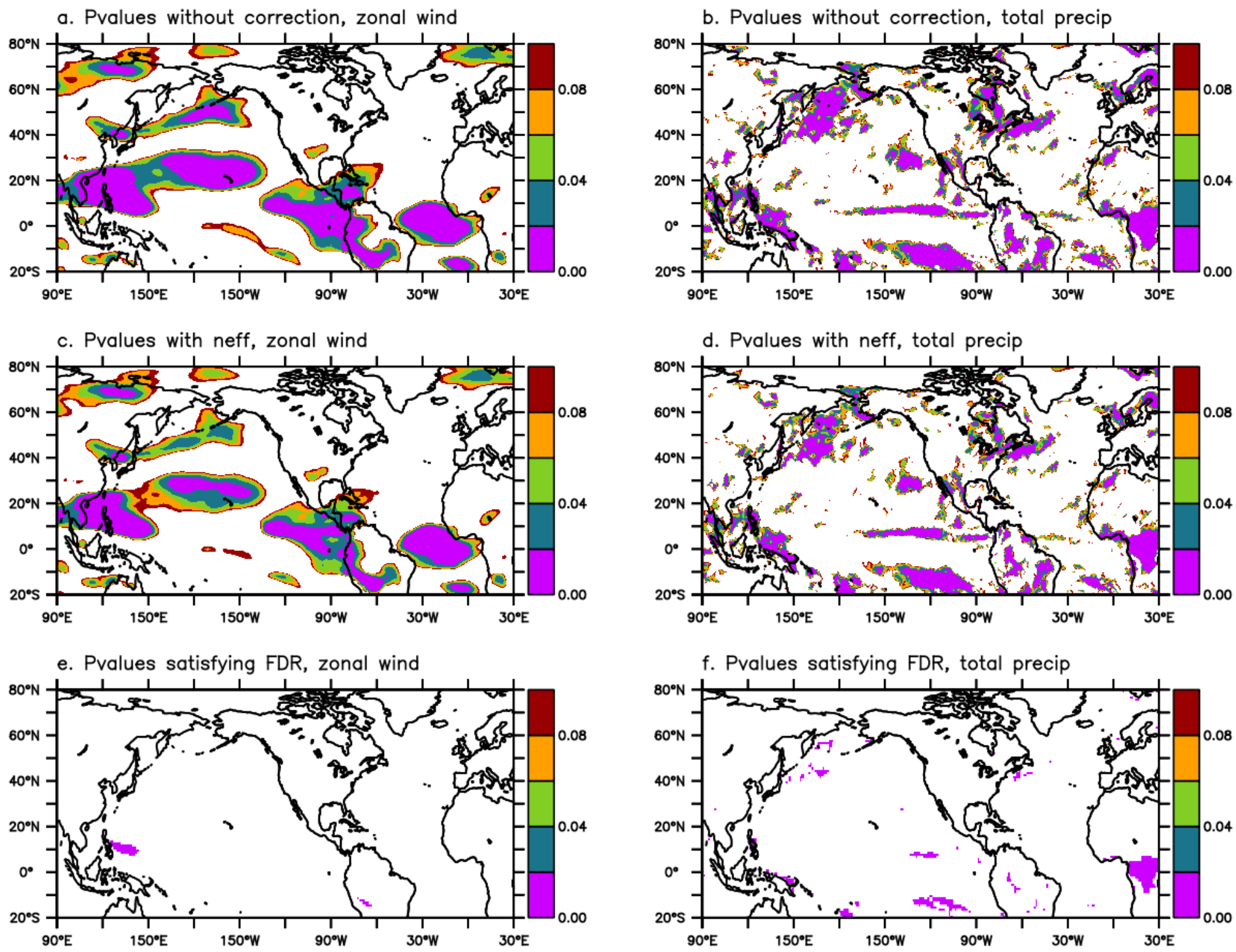

Fig. R1 Pvalues following two-sided t-tests applied to the 1979-2024 trends of the (left panels) vertically-averaged zonal wind between 300 hPa and 800 hPa, (right panels) total precipitation. (Upper panels) T-test with n_t=46 years, (middle panels) t-test with computation of an effective sample size neff, (lower panels) t-test with effective sample size n_{eff} and followed by the selection of the pvalues satisfying the FDR approach.

We have followed step by step the suggested corrections of the t-test. Concerning the dependence with time, we have computed the Lag-1 autocorrelation of the data, i.e the autocorrelation with a one-year lag, in order to get the effective sample size as

in Santer et al. (2000):

$$n_{eff} = n_t \frac{1-\rho_1}{1+\rho_1}, \quad (1)$$

where $n_t = 46$ (all the years from 1979 to 2024) and ρ_1 denotes the Lag-1 autocorrelation. The pvalues without and with that correction are shown in Figs.R1a-d for the zonal wind and total precipitation in ERA5. We do see some effects of that correction with some increase in the pvalues for the zonal wind in the North Pacific (compare panels a and c for instance).

Additionally, one should control for multiple testing, when testing every grid points independently as in Fig.1, Fig.4, Fig.5. With a significance level of 95%, you would expect 5% of the points to be significant, just by chance. when not controlling for multiple testing. One way to control for this is for example described by Wilks 2016 (DOI:10.1175/BAMS-D-15-00267.1).

In addition to the correction on the sample size, we have applied the correction on the spatial dependency of the tested variables following Wilks (2016). The results are shown in Figs. R1e-f. In that case, there are almost no regions where the pvalues are less than the global pvalue computed following the Wilks (2016)'s procedure. This result surprised us, in particular because the pvalues for the precipitation trends averaged over the PAC and ATL regions are equal to 2.2 10^{-5} and 5.5 10^{-5} respectively (those shown in Fig.2d) and are thus highly significant. To our knowledge, the two corrections (the effective sample size + FDR approach) have not been applied consecutively for the same test in previous studies in atmospheric sciences. We have thus some doubts about the relevance of applying both corrections consecutively because it treats the time and space dependencies as independent steps whereas we should apply a unique correction based on a spatio-temporal dependency as a whole. Because of the lack of coherence between the two corrections and because the trends of the spatially averaged precipitation in the PAC and ATL regions are highly significant, we have decided to only keep the correction on the sample size as in Santer et al. (2000). Concerning the statistical significance tests of the composites shown in Figures 4 and 5, we have applied a Student t test with the computation of an effective sample size where the autocorrelation is computed with a lag of 24 days which is the averaged time difference between two consecutive precipitation events. We have added a subsection in the Methods section to describe our statistical tests.

I expect some changes in the significance of some results, which would mean some rephrasing or re-interpretation of the results. I am not trying to be annoying or nitpicky, and I know that many studies in our fields are not as careful, but I do think it is very important, to avoid overstating confidence in results.

The revised figure 1 is shown in Fig. R2. There are some regions where considering the effective sample size has decreased the significance level both in the tropics and in the mid-latitudes. But this does not change our statements and the interpretation of the results. Finally, as a sanity check, we have also computed an equivalent to Fig. R2 by replacing the least squares linear regression of Fig. R2 by the Theil-Sen's slope estimator and the t-test by the non parametric Mann-Kendall test for monotonic trend (included in the pyMannKendall Python package). The advantage of the Theil-Sen's slope estimator compared to the least squares linear regression is that it is less

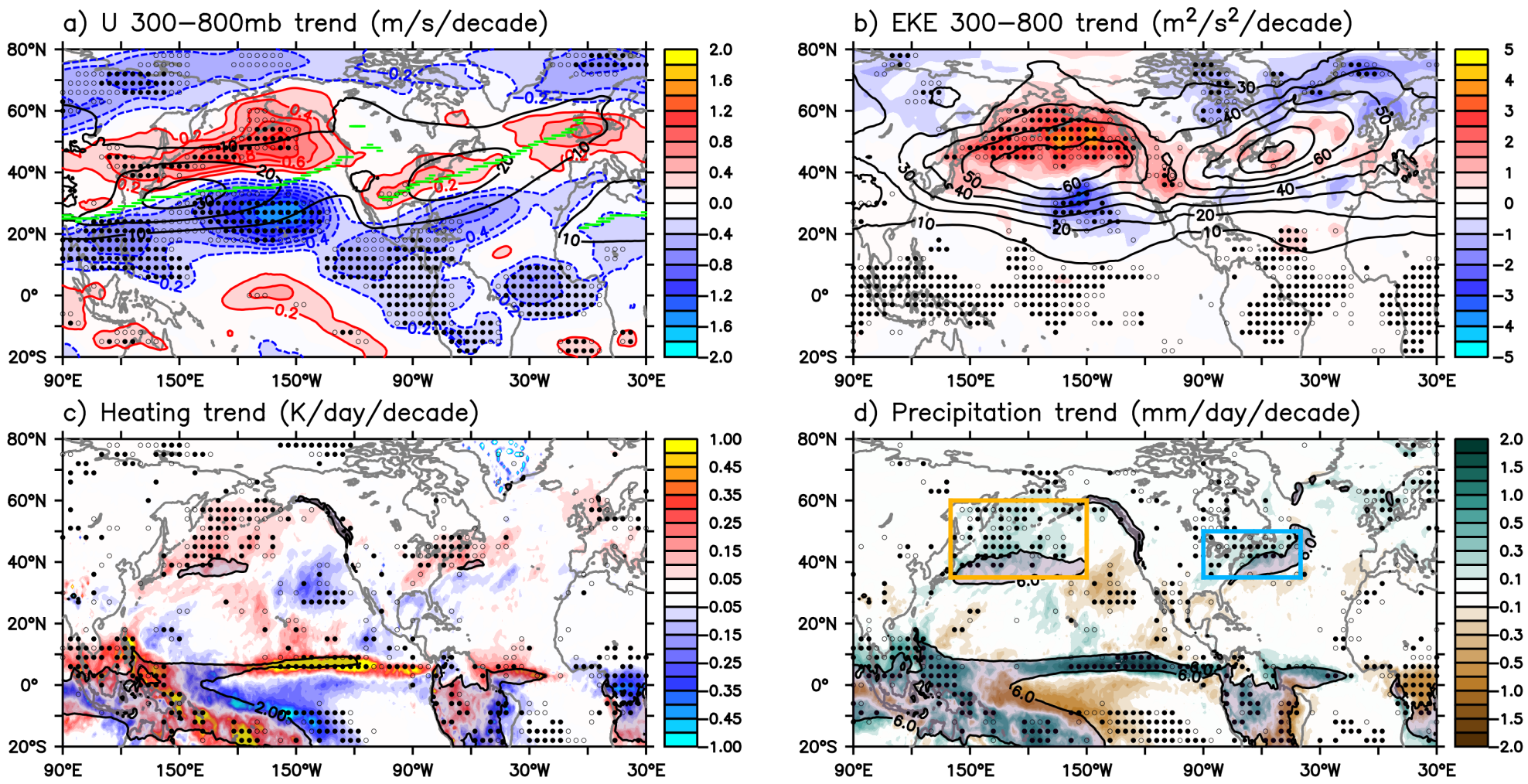

Fig. R2 Least squares linear regression trends during DJF from 1979 to 2024 using ERA5 reanalysis. a) 300-800 hPa zonal wind (int: 0.2 m s^{-1} $decade^{-1}$; red shadings for positive values and blue shadings for negative values) and climatology (contours). The green line corresponds to the climatological jet axis. b) 300-800 EKE (shadings; int: 0.5 m^2 s^{-2} $decade^{-1}$) and climatology (contours; int: 10 m^2 s^{-2}). c) 300-800hPa heating rate (shadings) and climatology (contours values greater than 2 K day^{-1}). d) total precipitation (shadings; units mm day^{-1} $decade^{-1}$) and climatology (black contour and shaded for values greater than 6 mm day^{-1}). Black dots and open circles indicate significance at the 5% and 10% levels respectively using a two-sided t-test.

sensitive to outliers. The results are shown in Fig. R3 and are quite close to Fig. R2. Some differences can be noticed however. For instance, in the tropics, the trend toward less precipitation in the middle of the Pacific (150°W-90°W; 20°S-0°S) is less eastward extended in the Theil-Sen's slope. But in the midlatitudes the differences in trends and significance are marginal. We have included this additional figure in the supplementary material to show the robustness of the results.

Finally about Fig5: Was the significance of the winds/geopotential tested? This seems important, as the argument rests on the Rossby wave propagating poleward.

The captions of Figs. 4 and 5 now indicate the fields to which the statistical test is applied, which was not the case in the initial version of the paper. In Fig. 5, the significance of the winds is tested on the right panels. It shows in particular that the acceleration of the North Atlantic jet is significant at 95% level 6 days after the precipitation events in the northwestern Pacific.

Minor comments

Introduction p.2 "This contradicts the sensitivity...", I find the word 'contradicts' too strong here. There are many factors that will ultimately determine the jet streams response as there are a multitude of (possible) mechanisms and drivers, so even if the models simulate a stronger tropical warming, it does not mean that the overall

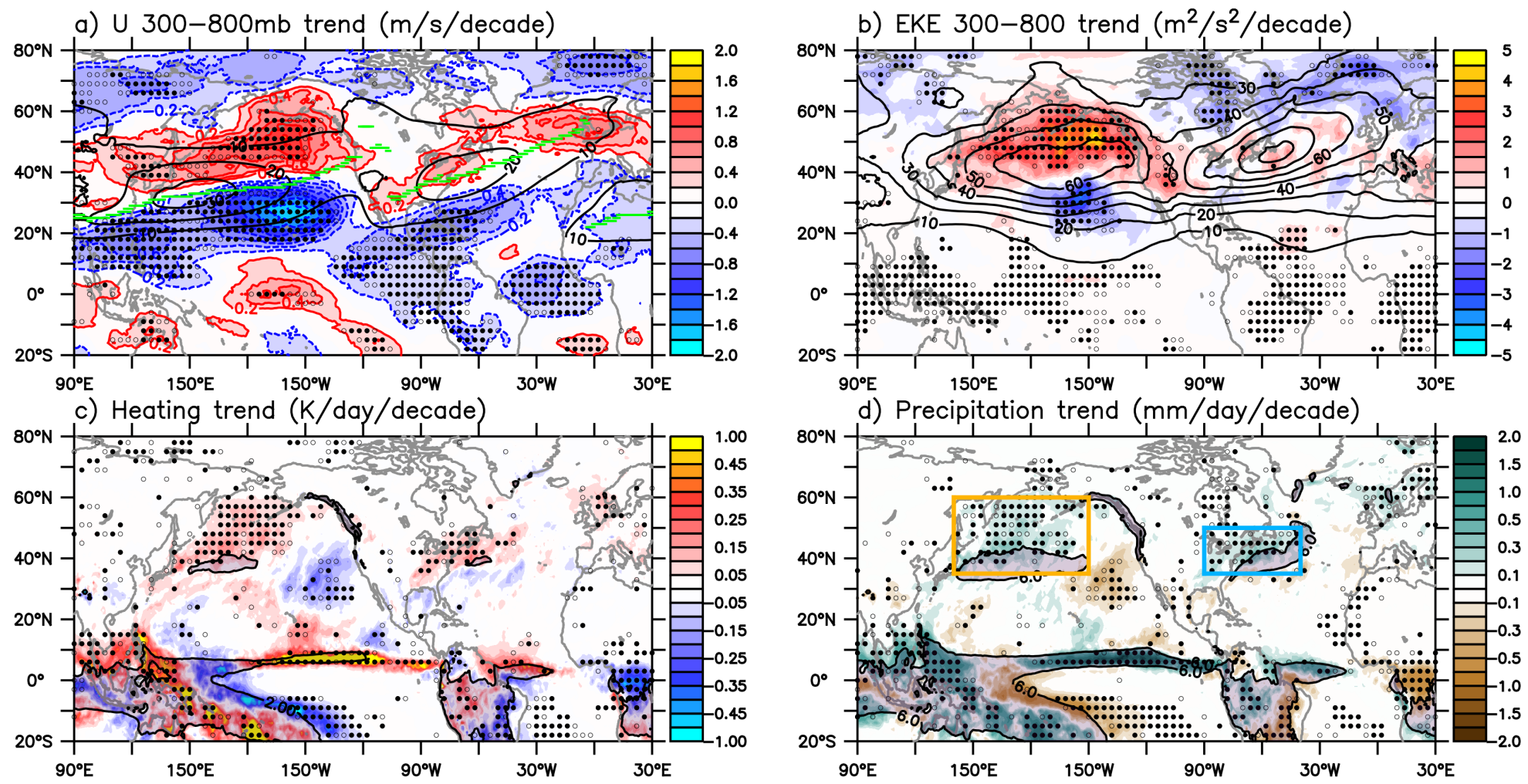

Fig. R3 Same as in Fig. R2 but using the Theil-Sen's slope estimator and the non parametric Mann-Kendall test for monotonic trend.

response has to be a stronger shift. While I agree with the sentiment of the statement, I would soften it.

"This contradicts the sensitivity ..." has been replaced by "It questions about the sensitivity".

p.2 "… obviously not for the same mechanistic reason…", Again I find this too strongly worded and not necessarily backed up by the references. Simpson et al. 2025 states in regard of the zonal mean jet shift in relation to tropical warming issue ' but that they may not have the correct relationship between upper tropospheric warming and this poleward shift, so this may be for the wrong reasons.'

"obviously not" has been suppressed and the sentence now reads like this: "Therefore although the models are able to qualitatively reproduce the zonal-mean observed jet streams trends, there is large intermodel spread [25] and the models do not necessarily reproduce the poleward shifts with the same intensity and the same mechanistic reasons as in the observations [1, 5].".

Chapter 2 Is there a reason why data from 2023 and 2024 was excluded?

There is no reason to exclude these years, it was just because the data have been initially downloaded until 2022. We have added the 2023 and 2024 years in all the figures of the revised paper.

Chapter 3 After reading the whole manuscript, I am a bit confused about the wording in the interpretation of the regression analysis here. Before reading all the way through, I had the impression that you were arguing that trends in the precipitation are driving the trends in the zonal winds due to phrases like "To conclude, the NPac jet trend is directly linked to NPac precipitation trend" or '…contribution of the PAC and ATL precipitation to the zonal wind trends can be estimated by …'. I was already prepared

to point out that this can not be concluded from the regression analysis, since the influence might be bi-directional or due to a common driver of both of them. After reading the whole manuscript I understand that you are arguing, that indeed trends in the tropics are the ultimate drivers of these trends (at least in parts). Maybe it is just me, but I would rephrase some parts of this chapter to make sure that it is not misunderstood to be seen as causal.

Indeed, the regression analysis of chapter 2 does not provide causal relationship. To make the text clearer, after "To conclude, the NPac jet trend is directly linked to NPac precipitation trend" we have added the following sentence "Of course, such a regression analysis does not provide causal relationship, it is just there to show how much of the zonal wind trends are accompanied by precipitation trends in various regions. Mechanistic arguments will be provided later in section 5.".

Fig.1: I would be consistent between subfigures regarding if shading or contours are used for climatology and trends respectively

In the new version of the figure, all trends are shown in shadings and all climatologies in contours (see Fig. 1 of the revised paper, or equivalently, Fig. R2 of the present reply document).

Technical

compage → compare Line numbers would be helpful

done

Reviewer 2 (Remarks to the Author):

The paper is clearly written and presents a plausible mechanism that could explain observed recent trends in jet streams whose cause is uncertain. Explaining these trends is a significant topic in the climate/dynamics. The results are not conclusive in the sense that the authors do not state that the mechanism presented is responsible for the observed trends, merely that it is a candidate mechanism for which they present some evidence. This is to be expected given the uncertainties involved.

I have one major methodological question:

The Ipac and Iatl indices are selected to be in regions where there are large trends. These are then regressed against the zonal jets which also have trends. Finally the regression coefficients and trends from the Ipac and Iatl indices are used to calculate the "trend congruent with" the zonal winds. A question that immediately arises is, how much of regression coefficient between the winds and rainfall indices is simply due to the fact that both have trends - to address this a suggestions would be to calculate the regression coefficients again after detrending both datasets.

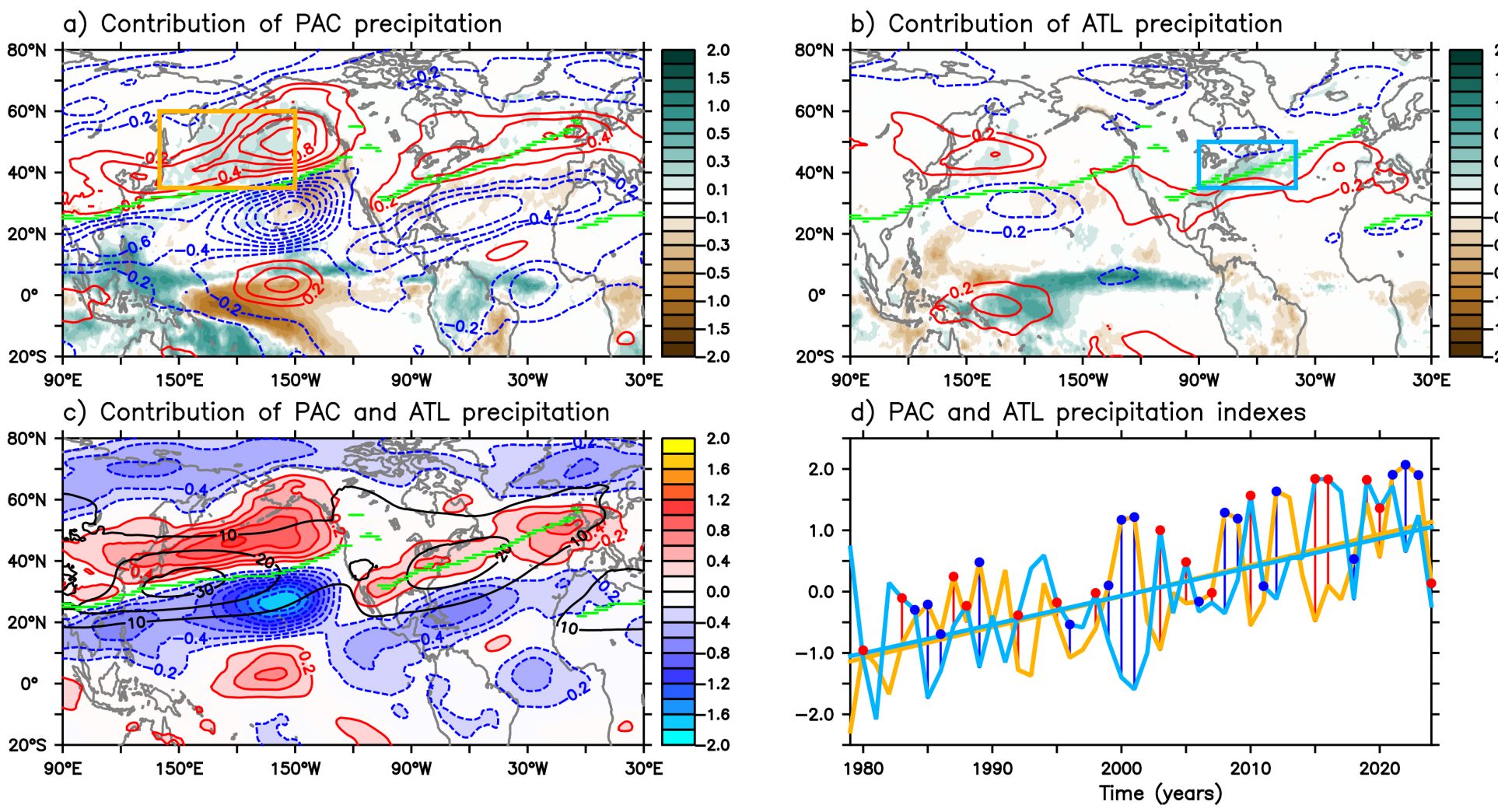

Fig. R4 Zonal wind and precipitation linear trends congruent with mid-latitude precipitation trends over two specific regions in the NPac and NAtl and relationship with ENSO. a) Contribution of PAC precipitation (orange box) to u (contours) and precipitation (shading) trends. b) Same as a) but for ATL precipitation (blue box). c) same as a) but using the PAC and ATL multilinear regression. d) PAC and ATL indexes as function of time. Blue and red dots correspond to Nino34 index greater than 1 and less than -1, respectively. The green line corresponds to the climatological jet axis.

Following reviewer's remark, we have computed the regression coefficients with detrended data. For instance, in Fig. R5a, we have computed the regressions by first detrending the data for the precipitation and zonal wind. The estimated detrended zonal wind obtained from the regression onto the index based on detrended

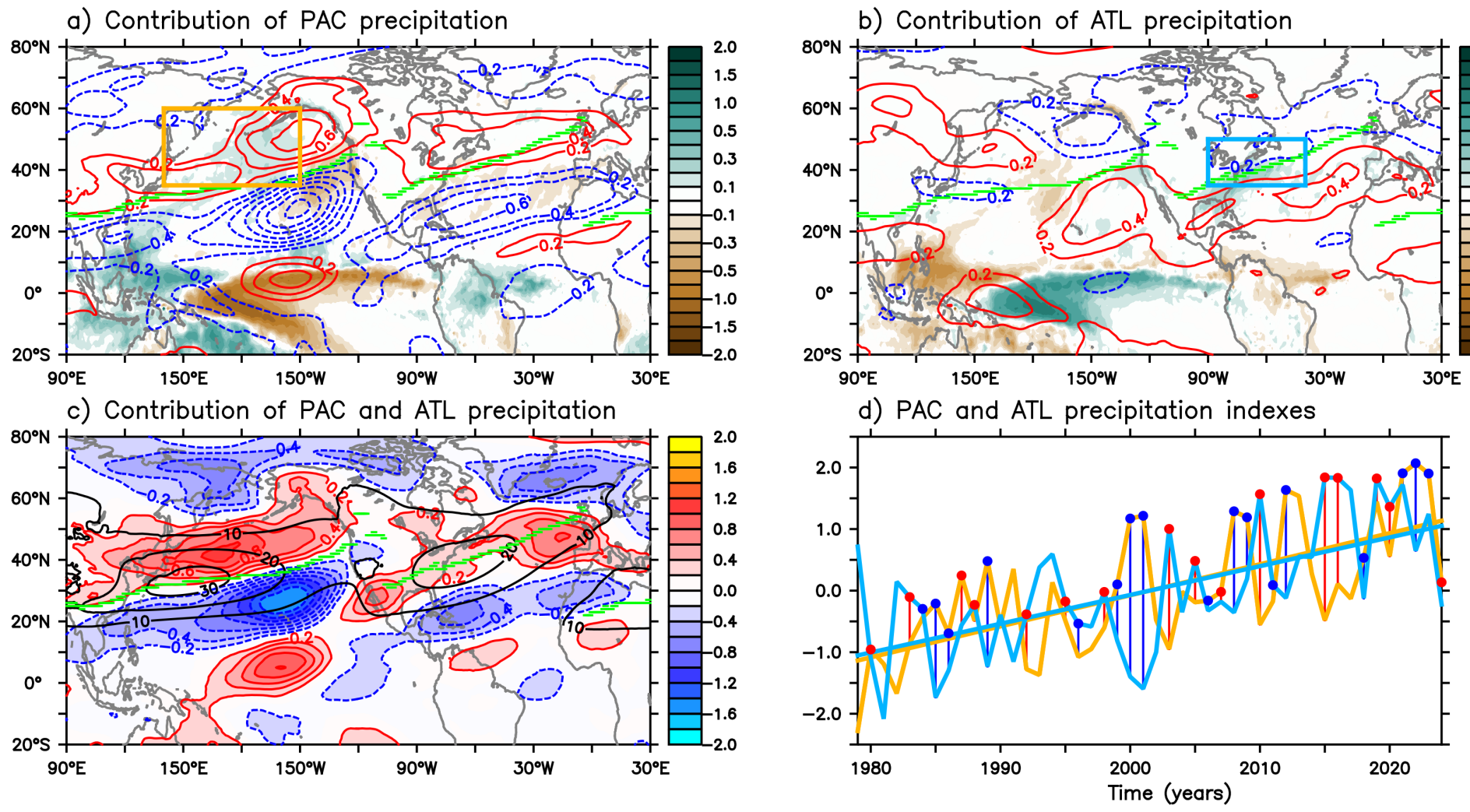

Fig. R5 Same as Fig. R4 but with detrended data.

precipitation can be written as

$$\hat{u^d}_{PAC} = \beta^{u^d}_{0PAC} + \beta^{u^d}_{1PAC}\frac{P^d_{PAC} - \overline{P^d_{PAC}}}{std(P^d_{PAC})} \tag{2}$$

where $\beta^{u^d}_{0PAC}$ and $\beta^{u^d}_{1PAC}$ denote the regression coefficients and P^d_{PAC} the averaged detrended precipitation in the PAC region. Then we multiply the slope of the regression by the trend of the precipitation index that is $\beta^{u^d}_{1PAC}\mathcal{T}(I_{PAC})$. When comparing the two regression coefficients for PAC, that are $\beta^{u}_{1PAC}\mathcal{T}(I_{PAC})$ (Fig. R4a) and $\beta^{u^d}_{1PAC}\mathcal{T}(I_{PAC})$ (Fig. R5a), we do see they resemble each other very well and the coefficient correlation is 0.96. It shows that the relationship between u and I_{PAC} holds at interannual time scales.

For ATL region, the similarity is less striking than for the PAC region but still there and the correlation is 0.61 (compare Fig. R4b with R5b). When combining the two regressions on the two indexes, we found a correlation of 0.89 and the two patterns resemble to each other very well (compare Fig. R4c with R5c). However we prefer to keep the formula without detrending the data in the main paper because, to our knowledge, Eq. (2) does not allow to write an equation relating $\beta^{u^d}_{1PAC}\mathcal{T}(I_{PAC})$ with any estimated trend in u. In contrast, our formulation without detrending the data allows to relate the estimated zonal wind trend from the regression on the PAC precipitation with the trend in PAC precipitation. Indeed, the following equation

$$\hat{u}_{PAC} = \beta^{u}_{0PAC} + \beta^{u}_{1PAC}I_{PAC} = \beta^{u}_{0PAC} + \beta^{u}_{1PAC}\frac{P_{PAC} - \overline{P_{PAC}}}{std(P_{PAC})} \tag{3}$$

leads to

$$\mathcal{T}(\hat{u}_{PAC}) = \beta^{u}_{1PAC}\mathcal{T}(I_{PAC}) \tag{4}$$

Minor Comments: - discussion of rainfall datasets - suggest an additional sentence or so discussing what rainfall observations are assimilated into ERA5 and any implications for the two specific regions selected.

As mentioned in Hersbach et al. (2020), radar and gauge precipitation data are only assimilated over the US and there is apparently no assimilation of precipitation in other regions. The total precipitation is the sum of convective and large-scale precipitation associated with the convection and large-scale clouds schemes of the IFS model. This information is provided in the Methods/data section.

- Comparison of Fig 1a and Fig 2a - this is a bit awkward as they are not in the same figure. Suggest strengthening the assertion that they are similar with a pattern correlation value.

The correlation value between Fig 1a and Fig 2a of the revised version is 0.91. This is now mentioned in the text. When considering the ATL region, it is only 0.48. and when considering both regions, the correlation reaches 0.97.

- Fig 4, 5: Significance at 2% is indicated by black dots - since there are multiple fields show in each panel it is unclear which is significant. If not all fields are significant how does this effect the interpretation of results.

The information was indeed lacking. Now the captions of the figures indicate to which field the significance test is applied.

Reviewer 3 (Remarks to the Author):

Review of Rivière et al., "Tropical origins of the recent trends in Northern Hemisphere wintertime jet stream"

Rivière et al. present a well-conceived study that provides a novel and compelling explanation for the observed trends in Northern Hemisphere wintertime jet streams over recent decades. As the authors note, most previously proposed mechanisms explaining the jet response to global warming are framed in zonal-mean terms, where tropical upper-tropospheric warming or Arctic amplification are considered the primary drivers of change. In contrast, the present manuscript proposes a mechanism in which asymmetric trends in tropical Pacific precipitation force a poleward displacement of the North Pacific (NPAC) jet and an acceleration of the North Atlantic (NATL) jet. Overall, the methods are sound and the proposed mechanism plausible. I find the manuscript worthy of publication. However, I would like the authors to clarify a few points before publication.

(1) The main conclusion of the manuscript is that asymmetric changes in tropical precipitation are related to the historical jet trends. The proposed causal chain is that tropical precipitation anomalies force a Rossby wave response that accelerates the poleward flank of the jet. In addition, the authors argue that these anomalies induce enhanced precipitation, increased cyclogenesis, and wave breaking at the western North Pacific boundary, which further reinforces the poleward displacement of the jet.

However, in Figure 1 the authors quantify how midlatitude precipitation anomalies (orange boxes) contribute to the jet trends rather than directly quantifying the contribution of tropical precipitation anomalies, which are central to the proposed mechanism. The authors could consider applying a lagged version of their regression framework using tropical precipitation as a predictor. Such an analysis would provide a more direct link between tropical anomalies and jet trends and would strengthen the evidence supporting the mechanism described.

Following the reviewer's suggestion, we have defined two precipitation-based indexes in the tropics, one over the Maritime Continent (yellow box in Fig. R6a) and another over the Central Pacific (blue box in Fig. R6b) and plotted exactly the same figure as Figure 2 of the paper with these two new indexes. The regressions on these tropical indexes only partly resemble the regressions made on the mid-latitude ones (compare Figs.R6 and R4). For instance, the poleward-shifted and accelerated North Pacific jet is still visible in the new multiple linear regression based on the two tropical indexes (Fig. R6c). The fact that the regressions based on tropical predictors and those based on mid-latitude ones do not resemble so well is not so surprising because these regressions are based on seasonal means and do not represent the influence of the tropics at intraseasonal time scales. Figure 3 of the paper shows that, in addition to La Nina seasons, we may have an increase in the mid-latitude North Pacific precipitation during El-Nino seasons when the MJO is close to phase 5. Therefore, the influence of the tropics in triggering the mid-latitude North Pacific precipitation events is partly hidden when considering seasonal means. It does not mean however that the tropics fully explain the important increase in the mid-latitude North Pacific precipitation events during the last two decades and more detailed analysis of such

events will be necessary in future studies but this is beyond the scope of the current paper. To conclude, we have kept Figure 2 of the paper with mid-latitude precipitation-based precursors but we have changed a sentence in the conclusion to emphasize the importance of both La-Nina and MJO phase 5 in triggering the mid-latitude Pacific precipitation: "The increased mid-latitude precipitation in NPac is favored by La-Niña and MJO-phase-5 tropical precipitation patterns characterized by more precipitation over the Maritime Continent".

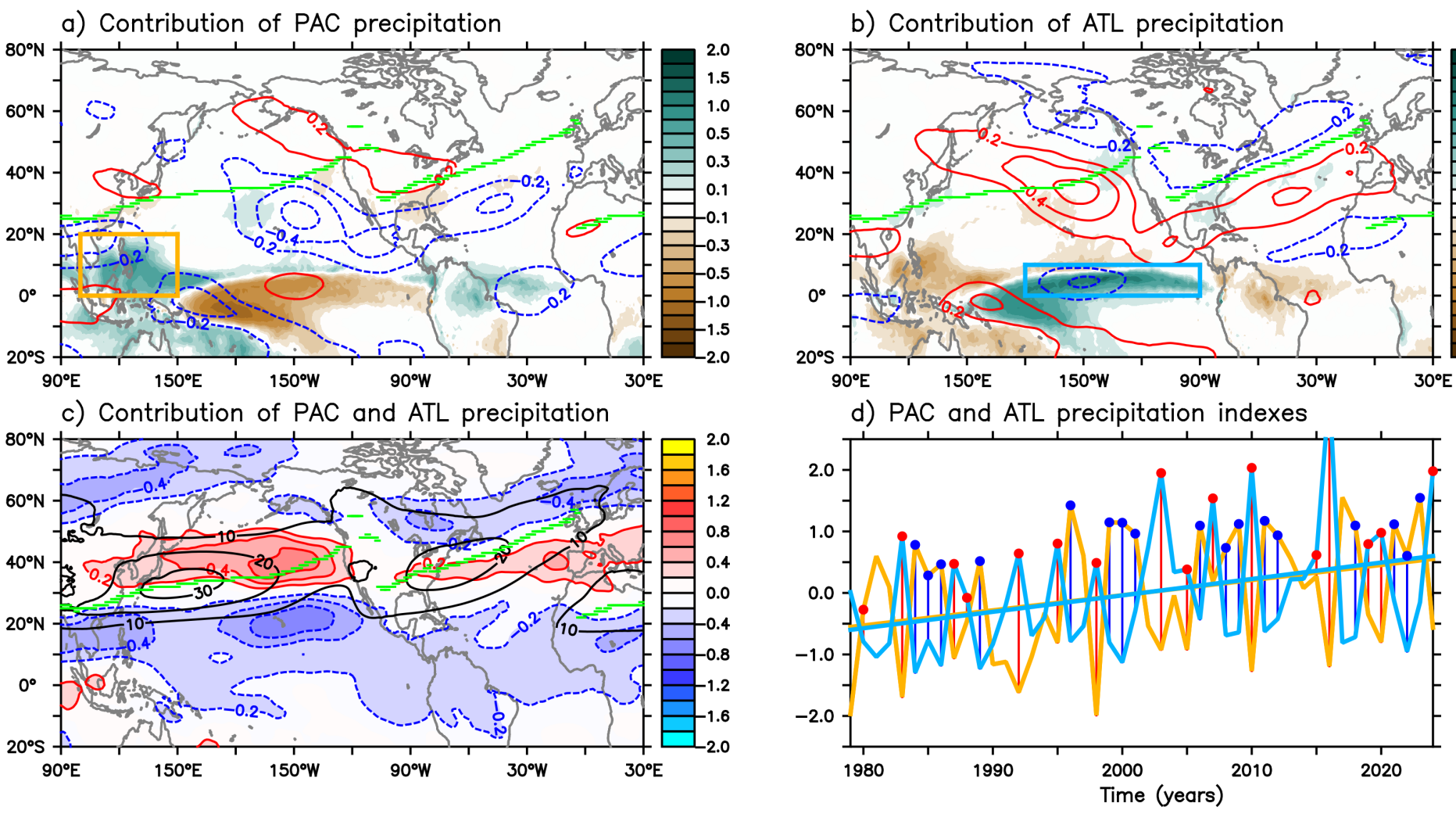

Fig. R6 As in Fig. R4 but for two predictors in the tropical region: one is based on the averaged precipitation over the Maritime Continent (yellow box in panel a) and the other on the averaged precipitation in the Central Pacific (blue box).

(2) In the introduction the author's state: "This contradicts the sensitivity of the jet poleward shift amplitude to the upper-tropospheric temperature gradient described above [18, 19]". I am not sure contradicts is the right terminology here since some papers have shown that the jet response to tropical warming not only depends on its magnitude but it strongly depends on the structure of the warming. See e.g., : Wang, S., Gerber, E. P., and Polvani, L. M. (2012). Abrupt circulation responses to tropical upper-tropospheric warming in a relatively simple stratosphere-resolving AGCM. Journal of Climate, 25(12), 4097–4115. https://journals.ametsoc.org/view/journals/clim/25/12/jcli-d-11-00166.1.xml

This statement was also found to be too strong by another reviewer. We have changed the sentence as follows: "This contradicts the sensitivity ..." has been replaced by "It questions about the sensitivity".

(3) Directly after the sentence I commented in point (2) the authors state: "Therefore although the models are able to qualitatively reproduce the observed jet streams trends…". This statement appears somewhat overconfident. Several studies have

shown that model agreement with observed jet trends depends strongly on the region, season, and wind level considered. For instance, model performance varies substantially between the North Pacific and North Atlantic basins and across different pressure levels. The authors may wish to moderate this statement or acknowledge this variability in model performance (see, e.g., Ossó et al. for a review).

The sentence has been changed as follows: "Therefore although the models are able to qualitatively reproduce the zonal-mean observed jet streams trends, there is large intermodel spread (Osso et al., 2025) and the models do not necessarily reproduce the poleward shifts with the same intensity and the same mechanistic reasons as in the observations (Woollings et al. 2023, Simpson et al. 2025)."

Authors' response

Reviewers' comments are in black, authors' answers in blue

We would like to thank the three reviewers for their constructive comments and suggestions

Reviewer 1

This is my second review of the manuscript and for the sake of discussion, I am waiving my anonymity.

I thank the authors for redoing the analysis so thoroughly. I am a bit surprised that the FDR approach reduced the significance as much as it did. Did the authors follow Wilks's suggestion to use an FDR significance level that is doubled to the base significance level to account for spatial correlation?

I am myself not fully sure what is the correct way to deal with significance testing on trends of spatially and temporally resolved fields, but to my understanding the two tests can be done consecutively, as one deals with the temporal correlation and the other with the problem of multiple testing. I have applied a similar approach, namely a pre-whitening and an FDR correction consecutively before (Fig. 5 or S1; https://doi.org/10.1038/s43017-025-00749-9). And I do believe that both things have to be accounted for.

However, I am not going to push the point further, since I know it is unconventional in the field and I further think the paper is well designed and written and is very interesting. But, if the authors are interested in discussing further, I am very keen to do so, since I have thought (and searched in the literature) about how to do testing on trends of spatially and temporally resolved fields several times now. This seems like it should be a standard tool in the field, shouldn't it?

I have no further comments on the manuscript and can recommend it for publication.

Philipp Breul

We would like to thank the reviewer for his new suggestions. But doubling the FDR significance level only leads to a slight increase in the size of the significant regions in the tropics but does not introduce new significant regions in the midlatitudes (Fig. R1). Another test we made but we did not show is to compute trends based on daily datasets rather than on seasonal mean datasets. Even though it does not affect the computed linear trends it has the effect to increase the size of the significant regions. Using daily datasets, some of the mid-latitude trends start to appear significant when both

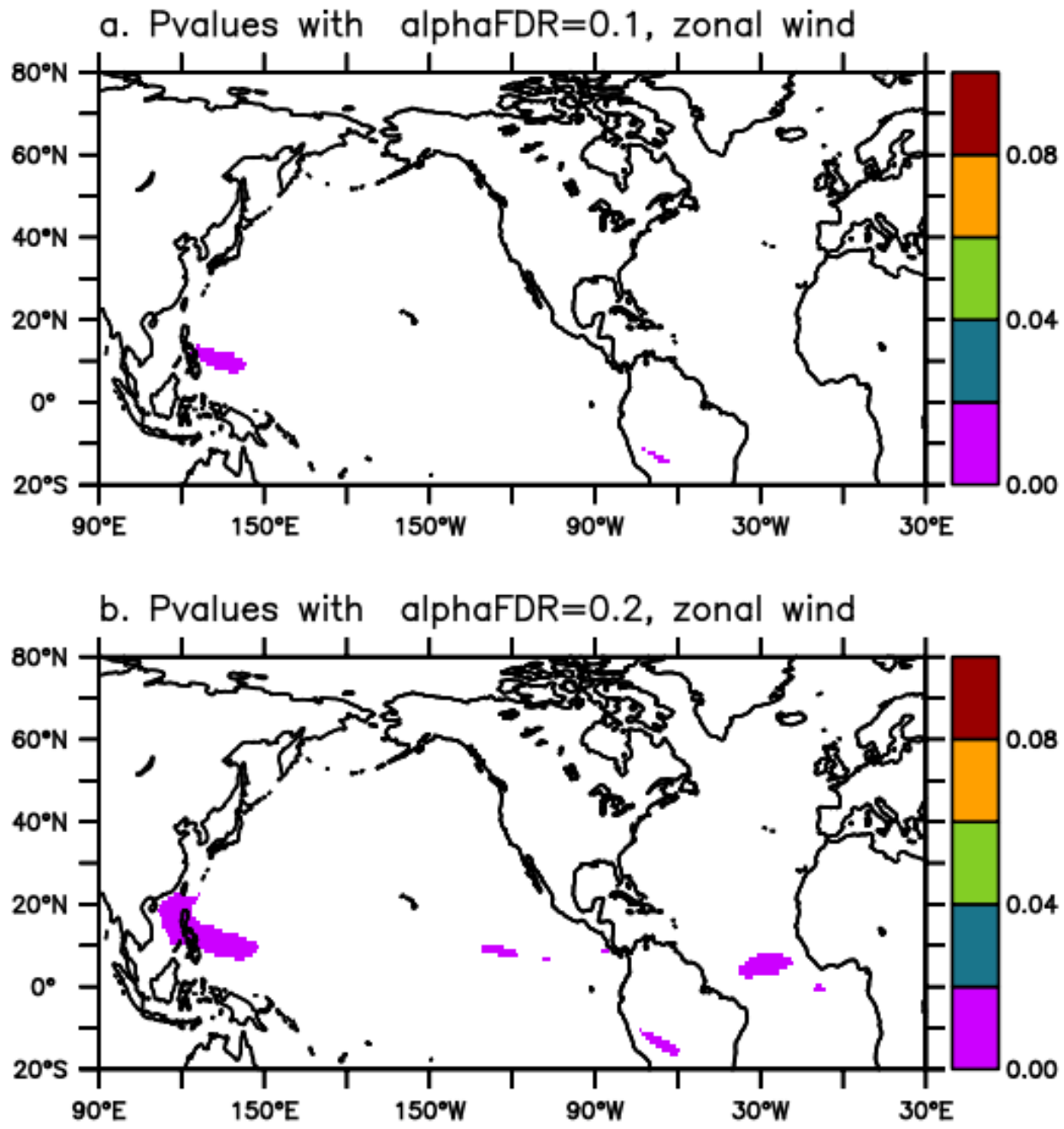

Fig. R1 Pvalues following two-sided t-tests following two corrections (sample size and FDR) applied to the 1979-2024 trends of the vertically-averaged zonal wind between 300 hPa and 800 hPa: (upper panel) $\alpha_{FDR} = 0.1$ and (lower panel) $\alpha_{FDR} = 0.2$.

corrections are used. But in our opinion, there is no reason to consider the daily-based trend more suitable than the seasonal-based one.

Reviewer 2 (Remarks to the Author):

The authors have satisfactorily addressed the concerns raised in review. Overall the paper is clearly written and presents a significant contribution to an important but uncertain topic in climate science. The methods are sound and reproducible, and the conclusion justified.

We would like to thank the reviewer for his/her relevant comments and the interesting discussions.

Reviewer 3 (Remarks to the Author):

The authors have carefully addressed my concerns. However,I do still have some reservations regarding the proposed mechanism. In particular, the regression maps with tropical precipitation are not conclusive and the causal direction of the relationships is not directly addressed by this study.

That said, although the mechanism is not definitely proven,the authors show enough evidence to considered it plausible and opens an interesting avenue for further investigation. Moreover, the authors have appropriately moderate the tone of their claims.

Overall, I find the article worthy of publication by Nature Communications.

We agree that the mechanism needs to be tested in different ways and that a better understanding of the processes leading to the heavy and sustained precipitation events at the entrance of the Pacific storm track is needed. Two sentences have been added in the conclusion: "The present study shows evidence of the link between precipitation anomalies and trends in the tropics and synoptic-scale events in the mid-latitude North Pacific. But circumstances under which such a link is valid need to be further investigated and the proposed mechanism needs to be tested in different ways, not only using reanalysis.". We would like to thank the reviewer for his/her helpful comments.
